# Populational analysis of the mortality-to-incidence ratio across 20 cancer groupings in the Russian Federation

**DOI:** 10.7189/jogh.16.04084

**Published:** 2026-03-13

**Authors:** Anastasiya Muntyanu, Amina Moustaqim-Barrette, Hibo Rijal, Vladimir Nechaev, Elena Pastukhova, Andrei Zubarev, James Logan, Ivan V Litvinov

**Affiliations:** 1 Division of Dermatology, University of Toronto, Toronto, Ontario, Canada; 2 Department of Experimental Medicine, McGill University, Montreal, Quebec, Canada; 3 Faculty of Medicine, McGill University, Montreal, Quebec, Canada; 4 Queen’s University School of Medicine, Kingston, Ontario, Canada; 5 Independent Consultant, Saint Petersburg, Russia; 6 Faculty of Medicine, University of Toronto, Toronto, Ontario, Canada; 7 Cancer Research Program, McGill University Health Centre Research Institute, Montreal, Quebec, Canada; 8 Geographic Information System (GIS), Ottawa, Ontario, Canada

## Abstract

**Background:**

Cancer represents a leading cause of death globally. The mortality to incidence ratio (MIR) may vary across populations, reflecting regional disparities in care. We aimed to determine MIR trends by cancer subtype across the 80 recognised jurisdictions of the Russian Federation between 2008 and 2018.

**Methods:**

We used publicly accessible data from the Pyotr Alexandrovich Herzen Moscow Oncology Research Institute from 2008–2018. We used descriptive and linear regression analyses to estimate MIR from crude mortality and incidence rates across geographical regions.

**Results:**

Between 2008 and 2018, surrounding adjacent jurisdictions were significantly associated with increased MIRs compared to centring cities. The culturally distinct region of the Republic of Adygea reported outliers in MIR for sigmoid, rectal, and anal cancers (MIR = 0.754; 95% confidence interval (CI) = 0.579, 0.983), lung cancer (MIR = 1.07 (95% CI undefined), breast cancer (MIR = 0.458; 95% CI = 0.341, 0.615), and melanoma (MIR = 0.462; 95% CI = 0.201, 1.059), which was not observed in adjacent predominantly Caucasian-populated/western Krasnodar krai that fully envelops the Republic geographically. Gross domestic product (11-year average), the number of physicians, and the number of oncologists per 10 000 people were significantly negatively associated with increased MIR (*P* < 0.05).

**Conclusions:**

Differences in MIR for cancer have been observed between capital cities and their surrounding jurisdictions, with increased ratios noted in demographically distinct regions such as the Republic of Adygea. This suggests that population-level differences in MIR and subsequent cancer outcomes are influenced by region, culture, and socioeconomic variations. These findings may inform public health policy on intersectional disparities in MIR across different populations in the Russian Federation, with applicability to other countries.

The International Agency for Research on Cancer’s (IARC) Global Cancer Observatory (GLOBOCAN) 2022 report shows a rise in new cancer cases per year from 14.1–20 million between 2012 and 2022 [1]. Annual deaths secondary to cancer have also increased from 8.2–10 million during the same period. The burden of cancer is further expected to increase worldwide due to the growth and ageing of the population [1]. Demographics-based predictions indicate a continued rise of annual cancer incidence to 35 million by 2050 [1]. In Russia, cancer ranks as the second leading cause of mortality, following cardiovascular diseases [2].

Cancer incidence and mortality vary between regions within a country, often reflecting regional health care disparities. Our prior studies investigated the incidence of different cancers in the Russian Federation [3–5]. On the other hand, the mortality-to-incidence ratio (MIR) is the proportion of deaths among new cases diagnosed. The ratio should thus range from zero (no deaths) to one (equal numbers of deaths and new cases). MIR can exceed the value ‘1’ in small populations or for highly lethal cancers due to statistical instability and delayed mortality. MIR compares crude mortality and incidence rates, and its value can serve as a proxy for 1 – survival [6]. MIR<1, MIR = 1, or MIR>1 indicates that fewer, equal to, or more people died from a particular cancer than had been diagnosed within that given year, respectively [7]. As such, MIR>1 may indicate an increase in mortality from possible sudden decreased survival for a given year (since incidence can predate mortality by years or decades), a decrease in incidence from reduced diagnoses or a combination of both measures [7]. MIR>1 may also indicate inconsistencies in coding for death certificates and cancer incidence records [8]. Importantly, it may also highlight autopsy-only diagnoses [8,9].

The objective of our study was to quantify MIRs across 20 cancer groups in 80 jurisdictions of the Russian Federation (2008–2018), to evaluate jurisdictional differences, and to explore associations with population-level indicators. We hypothesised that MIRs vary systematically between urban capital cities and their surrounding jurisdictions within the Russian Federation, and that MIRs would be negatively associated with regional socioeconomic and health care capacity (*e.g.* gross domestic product (GDP) *per capita*, physician and oncologist density).

## METHODS

### Study design

This study was completed using an ecological study design similar to our previous analyses [10]. The study design and methods for data reporting were conducted in accordance with the STROBE checklist (Appendix S1 in the **Online Supplementary Document**). As this study only used open-source government reports, it did not require institutional review board assessment/approval.

### Setting, participants, and data sources

We obtained crude incidence and mortality rates per 100 000 people per cancer grouping in the Russian Federation from publicly available reports prepared by the Herzen Moscow Oncology Institute for the years 2008–2018. The end point of 2018 was selected, as in the years following the global pandemic (*i.e.* 2019-year data was reported in the middle of the 2020 pandemic), and subsequent militarised geopolitical unrest had a significant impact on healthcare access and data reporting [11–13]. Several cancer groupings, including cancer of the meninges, brain, spinal cord, cranial nerves, and other parts of the central nervous system (CNS) (C70–72) and thyroid cancer (C73), had no reported data for the years 2008–2010.

We included the following cancer groupings in the study (listed according to the International Classification of Diseases (ICD) codes): C00–14, C15, C16, C18, C19–21, C22, C25, C32, C33-34, C40–41, C43, C44, C50, C53, C54, C56, C61, C67, C70–72, C73 (Table S1 in the **Online Supplementary Document**). No information was available regarding clinical staging of the disease, clinical/pathological subtype, or treatment approaches. Additionally, no individual patient data were available regarding demographics or other comorbidities.

The Russian Federation consists of eight federal districts that are conglomerates of internationally recognised federal subjects (*i.e.* individual jurisdictions). Federal subjects include oblasts, krais, autonomous republics, autonomous oblasts, and cities with special status (*i.e.* Moscow and Saint Petersburg). We extracted the data for each of the aforementioned categories.

### Variables and study size

We obtained data on healthcare indicators by federal district, federal subject, and the Russian Federation from the Federal Research Institute for Health Organisation and Informatics of the Ministry of Health of the Russian Federation, as well as from publicly available reports. Variables analysed included: the total number and the number per 10 000 individuals of physicians and oncologists (both paediatric and adult) and GDP over 11 years. These variables are populational and reported by jurisdiction. We included data from 80 jurisdictions within the Russian Federation.

### Statistical methods

We used SAS, version 9.4 (SAS Institute Inc., Cary, North Carolina, USA) and *R*, version 4.4.2 (R Core Team, Vienna, Austria) [14]. We created incidence and mortality plots and fitted them with linear regression curves. 95% confidence intervals (CIs) were reported, and regions of interest were identified. As previously described [8,15], we used average population estimates for the 10-year period and computed a period MIR (2008–2018) as the ratio of total deaths to total incident cases:

MIR = ∑ D/ ∑ C

We produced CIs for MIRs by calculating standard errors and log-transforming the MIR. However, no confidence intervals were generated for MIR>1, as the method yielded undefined confidence bounds due to an indeterminate form in the calculations. Associations between MIR and population-level factors were analysed using simple linear regression. *P*-values of <0.05 were considered statistically significant.

## RESULTS

### Summary statistics for the Russian Federation

The average MIR for all cancers in the Russian Federation from 2008–2018 was 0.518 (95% CI = 0.470, 0.571) (**Table 1**). Liver and intrahepatic cancer had the highest average MIR in the country (mean (x̄) = 1.17), with the highest regional MIR found in Moscow Oblast (x̄ = 2.00). The second-highest MIR across Russia was noted for pancreatic cancer (MIR = 0.975; 95% CI = 0.889, 1.07). Non-melanoma skin cancers had the lowest MIRs nationwide (MIR = 0.024; 95% CI = 0.004, 0.149).

**Table 1:** MIR and 95% CIs for each cancer type based on select jurisdictions, with comparisons between urban centres (*i.e.* Moscow city and Saint Petersburg) to the surrounding region (*i.e.* Moscow Oblast and Leningrad Oblast) or between the Republic of Adygea and the surrounding Krasnodar krai during 2008–2018

Cancer type	Jurisdictions, MIR (95% CI)						
	**Moscow City**	**Moscow Oblast**	**Saint Petersburg**	**Leningrad Oblast**	**Republic of Adygea**	**Krasnodar Krai**	**Russian Federation**
Malignant neoplasms of the lip, oral cavity, and pharynx (C00–14)	0.699 (0.447, 1.092)	0.722 (0.488, 1.067)	0.676 (0.447, 1.022)	0.697 (0.472, 1.031)	0.762 (0.540, 1.076)	0.545 (0.327, 0.908)	0.580 (0.355, 0.948)
Oesophageal cancer (C15)	1.057*	1.041*	0.949 (0.787, 1.144)	1.077*	1.107*	0.853 (0.549, 1.326)	0.863 (0.625, 1.191)
Stomach cancer (C16)	0.936 (0.842, 1.040)	1.011*	0.859 (0.744, 0.990)	0.989 (0.950, 1.029)	1.050*	0.808 (0.660, 0.989)	0.812 (0.678, 0.973)
Colon cancer (C18)	0.715 (0.570, 0.898)	0.759 (0.611, 0.942)	0.649 (0.516, 0.817)	0.737 (0.583, 0.931)	0.686 (0.507, 0.927)	0.552 (0.395, 0.771)	0.586 (0.417, 0.823)
Rectosigmoid junction, rectum, and anal cancer (C19–21)	0.685 (0.493, 0.953)	0.707 (0.522, 0.957)	0.615 (0.447, 0.848)	0.690 (0.508, 0.937)	0.754 (0.579, 0.983)	0.521 (0.347, 0.781)	0.604 (0.417, 0.875)
Liver and intrahepatic bile duct cancer (C22)	1.929*	2.000*	1.149*	1.733*	1.500*	1.423*	1.168*
Pancreatic cancer (C25)	1.264*	1.385*	1.042*	1.343*	1.106*	0.927 (0.792, 1.084)	0.975 (0.889, 1.071)
Laryngeal cancer (C32)	0.667 (0.300, 1.484)	0.750 (0.437, 1.286)	0.634 (0.304, 1.323)	0.750 (0.437, 1.286)	0.698 (0.399, 1.222)	0.600 (0.293, 1.227)	0.632 (0.323, 1.237)
Tracheal, bronchus, and lung cancer (C33–34)	1.047*	1.085*	0.922 (0.842, 1.011)	1.144*	1.070*	0.863 (0.765, 0.974)	0.853 (0.753, 0.966)
Cancer of the bone articular cartilage (C40–41)	1.000 (1.000, 1.000)	1.000 (1.000, 1.000)	0.818 (0.339, 1.974)	0.750 (0.267, 2.107)	1.091*	0.846 (0.407, 1.761)	0.799 (0.324, 1.971)
Malignant melanoma of the skin (C43)	0.453 (0.207, 0.995)	0.371 (0.142, 0.974)	0.414 (0.197, 0.869)	0.391 (0.148, 1.028)	0.462 (0.201, 1.059)	0.291 (0.102, 0.826)	0.365 (0.128, 1.036)
Skin cancer (not including melanoma) (C44)	0.030 (0.005, 0.189)	0.032 (0.005, 0.184)	0.040 (0.008, 0.201)	0.042 (0.008, 0.225)	0.022 (0.005, 0.091)	0.021 (0.005–0.099)	0.024 (0.004, 0.149)
Breast cancer (C50)	0.371 (0.269, 0.512)	0.365 (0.261, 0.511)	0.413 (0.310, 0.551)	0.395 (0.281, 0.557)	0.458 (0.341, 0.615)	0.342 (0.238, 0.491)	0.340 (0.234, 0.493)
Cervical cancer (C53)	0.532 (0.326, 0.868)	0.397 (0.228, 0.692)	0.531 (0.342, 0.825)	0.527 (0.343, 0.810)	0.552 (0.369, 0.826)	0.417 (0.254, 0.685)	0.386 (0.228, 0.653)
Ovarian cancer (C56)	0.729 (0.544, 0.977)	0.671 (0.481, 0.936)	0.624 (0.453, 0.859)	0.690 (0.496, 0.960)	0.607 (0.411, 0.897)	0.509 (0.321, 0.806)	0.546 (0.355, 0.841)
Prostate cancer (C61)	0.274 (0.184, 0.408)	0.370 (0.248, 0.550)	0.407 (0.285, 0.581)	0.488 (0.344, 0.691)	0.455 (0.318, 0.652)	0.285 (0.184, 0.442)	0.359 (0.234, 0.552)
Bladder cancer (C67)	0.469 (0.239, 0.919)	0.543 (0.302, 0.976)	0.442 (0.234, 0.834)	0.562 (0.314, 1.004)	0.562 (0.329, 0.959)	0.393 (0.193, 0.801)	0.433 (0.215, 0.873)
Cancer of the meninges, brain, spinal cord, cranial nerves, and other parts of the central nervous system (C70–72)†	1.326*	1.333*	0.877 (0.677, 1.135)	1.302*	1.100*	0.889 (0.674, 1.172)	0.876 (0.637, 1.205)
Thyroid cancer (C73)†	0.116 (0.015, 0.910)	0.109 (0.012, 0.998)	0.091 (0.015, 0.540)	0.110 (0.019, 0.641)	0.112 (0.021, 0.605)	0.057 (0.007, 0.477)	0.103 (0.012, 0.860)
All cancers combined	0.589 (0.540, 0.642)	0.623 (0.575, 0.676)	0.588 (0.544, 0.636)	0.691 (0.643, 0.742)	0.546 (0.499, 0.598)	0.459 (0.414, 0.508)	0.518 (0.470, 0.571)

C – cancer based on the International Classification of Diseases descriptive coding, CI – confidence interval, MIR – mortality to incidence ratio

*MIR ≥ 1 typically occurs in small populations or high-lethality cancers and can reflect instability.

†Data included from 2010–2018.

### Regional differences across the Russian Federation

Of the individual regions, Leningrad Oblast demonstrated some of the highest MIRs for several cancers that have effective screening strategies as well as for those that do not (including oesophageal, gastric, colon, liver and intrahepatic, pancreatic, trachea/bronchi/lung, bone/cartilage, and ovarian cancers (**Table 1**; Figure S1 in the **Online Supplementary Document**). Leningrad Oblast had the highest MIR for all cancers combined in the country (MIR = 0.691; 95% CI = 0.643, 0.742), a level significantly higher than that of the neighbouring federal city of Saint Petersburg. Following Leningrad Oblast were Vladimir Oblast and Kemerovo Oblast (Figures S3 and S4 and Table S2 in the **Online Supplementary Document**). Other regions (Krasnoyarsk – outliers in MIR for liver, pancreas, lung cancers, Smolensk – oral and oesophagus; Vologda – oesophagus and stomach cancers) showed notable findings (Figures S5–7 in the **Online Supplementary Document**).

**Figure 1:**
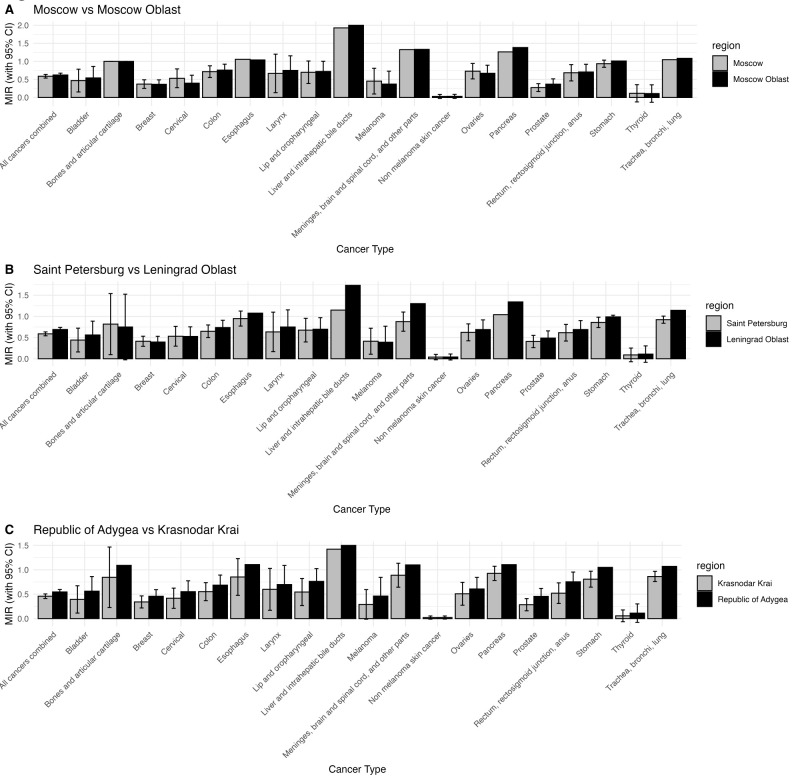
Comparison of MIR across cancer types. **Panel A.** Moscow City *vs.* Moscow Oblast. **Panel B.** Saint Petersburg *vs.* Leningrad Oblast. **Panel C.** Krasnodar krai *vs.* the Republic of Adygea

With respect to the capital cities of Moscow and Saint Petersburg (**Figure 1**, Panels A and B) and their surrounding regions, Saint Petersburg had a higher MIR than expected for breast cancer (MIR = 0.413; 95% CI = 0.309, 0.551) and melanoma (MIR = 0.414; 95% CI = 0.197, 0.869) (**Table 1**). In 16 of 20 cancer groupings, Leningrad Oblast had higher MIRs than Saint Petersburg city. Similarly, Moscow Oblast had a higher MIR than Moscow City in 12 of 20 cancer groupings considered (**Figure 1**, Panels A and B). Moscow Oblast had a high MIR for gastric, colon, pancreatic, and meninges/brain/spinal cord cancers (Figure S2 in the **Online Supplementary Document**). In Moscow city, outliers with high MIR were observed for colon, pancreatic, ovarian, CNS, and melanoma (**Figure 1**, Panel A; Figure S8 in the **Online Supplementary Document**). For melanoma, Moscow Oblast had a much lower MIR than Moscow city, though overall Moscow Oblast had a higher MIR than Moscow city; both remained higher than the national average.

The Republic of Adygea was highlighted by the analysis for having several outlying cancers with MIRs significantly higher than expected. Several of these malignancies can be identified with early screening and are treatable, including sigmoid, rectal, and anal cancers, lung cancer, breast cancer, and melanoma (Figure S9 in the **Online Supplementary Document**). Other cancers identified through screening, such as lip and oropharyngeal as well as cervical cancer, also had high MIRs in the Republic of Adygea, but were not outliers on the curve. Adygea is geographically surrounded by the Krasnodar krai, where we do not observe significant outliers, and this region ranks among the lowest 10 jurisdictions for overall MIRs across the country (**Figure 1**, Panel C).

Several regions were consistently found to have lower MIRs for most cancer types, including the Republic of Ingushetia, the Chechen Republic, and the Chukotka Autonomous Okrug (Figures S1–9 in the **Online Supplementary Document**). As mentioned in the methods, we lacked data from 2008–2010 for CNS and thyroid cancers. While these cancer groups did appear as outliers (notably, CNS showed high MIR in Moscow City and Leningrad Oblast), these findings should be interpreted with caution. Smaller jurisdictions, including the Republic of Ingushetia, the Chechen Republic, and the Chukotka Autonomous Okrug, showed consistently low MIRs, which may be related to population size and potential under-ascertainment.

### Risk factors analysis

When analysing risk factors for the Russian Federation as a whole, GDP (11-year average) had a significant negative association with the following cancers: breast cancer (*P* = 0.003), prostate cancer (*P* < 0.0001), bladder cancer (*P* = 0.026), and thyroid cancer (*P* < 0.001) (**Table 2**). For most other cancers, there were statistically nonsignificant negative associations with GDP, while melanoma, lip, and oropharyngeal cancer, oesophageal, pancreatic, tracheal, bronchus, and lung cancers showed positive trends.

**Table 2:** Univariate linear regression results for cancer MIRs by 11-year average GDP, number of physicians per 10 000 people, and number of oncologists per 10 000 people

Cancer type	GDP (11-year average), coefficient (95% CI)	*P*-value	Physicians per 10 000 people, coefficient (95% CI)	*P*-value	Oncologists per 10 000 people, coefficient (95% CI)	*P*-value
Malignant neoplasms of the lip, oral cavity, and pharynx (C00-14)	6.88E-08 (–4.36E-09, 1.42E-07)	0.065	–2.19E-04 (–2.95E-03, 2.51E-03)	0.874	–3.76E-03 (–1.18E-01, 1.11E-01)	0.948
Oesophageal cancer (C15)	6.89E-08 (–1.57E-08, 1.54E-07)	0.109	2.69E-04 (–2.87E-03, 3.41E-03)	0.865	–7.86E-02 (–2.09E-01, 5.20E-02)	0.235
Stomach cancer (C16)	–4.52E-08 (–1.19E-07, 2.86E-08)	0.227	–2.64E-03 (–5.30E-03, 2.11E-05)	0.0518	–1.63E-01 (–2.71E-01, –5.42E-02)	0.004
Colon cancer (C18)	–3.17E-08 (–1.01E-07, 3.79E-08)	0.368	–2.80E-03 (–5.29E-03, –3.11E-04)	0.028	–1.28E-01 (–2.32E-01, –2.42E-02)	0.0162
Rectosigmoid junction, rectum, and anal cancer (C19–21)	–5.38E-08 (–1.12E-07, 4.70E-09)	0.071	–2.33E-03 (–4.45E-03, –2.05E-04)	0.032	–1.56E-01 (–2.41E-01, –7.07E-02)	<0.001
Liver and intrahepatic bile duct cancer (C22)	–2.15E-08 (–2.31E-07, 1.88E-07)	0.839	–9.42E-03 (–1.68E-02, –2.03E-03)	0.013	–1.94E-01 (–5.13E-01, 1.24E-01)	0.229
Pancreatic cancer (C25)	5.37E-09 (–9.39E-08, 1.05E-07)	0.915	–3.05E-03 (–6.62E-03, 5.15E-04)	0.093	–9.15E-02 (–2.42E-01, 5.94E-02)	0.231
Laryngeal cancer (C32)	–8.63E-09 (–9.76E-08, 8.03E-08)	0.071	–1.84E-03 (–5.07E-03, 1.39E-03)	0.26	–1.43E-01 (-2.76E-01, –9.85E-03)	0.036
Tracheal, bronchus, and lung cancer (C33–34)	1.03E-08 (–6.83E-08, 8.89E-08)	0.795	–2.02E-03 (–4.87E-03, 8.17E-04)	0.16	–1.35E-01 (–2.52E-01, –1.80E-02)	0.024
Cancer of the bone articular cartilage (C40–41)	–5.49E-08 (–2.30E-07, 1.21E-07)	0.535	–8.70E-04 (–7.29E-03, 5.55E-03)	0.788	–9.65E-02 (–3.65E-01, 1.72E-01)	0.477
Malignant melanoma of the skin (C43)	1.83E-08 (–2.43E-08, 6.09E-08)	0.396	1.70E-03 (1.82E-04, 3.22E-03)	0.029	–3.63E-02 (–1.01E-01, 2.88E-02)	0.271
Skin cancer (not including melanoma) (C44)	–9.68E-11 (–9.77E-09, 9.57E-09)	0.984	2.52E-05 (–3.28E-04, 3.79E-04)	0.887	–9.77E-03 (–2.44E-02, 4.91E-03)	0.189
Breast cancer (C50)	–5.21E-08 (–8.63E-08, –1.78E-08)	0.003	–2.09E-03 (–3.33E-03, –8.52E-04)	0.0012	–6.64E-02 (–1.20E-01, –1.30E-02)	0.015
Cervical cancer (C53)	–4.58E-08 (–1.05E-07, 1.32E-08)	0.127	–1.01E-03 (–3.18E-03, 1.17E-03)	0.36	–4.04E-02 (–1.32E-01, 5.09E-02)	0.382
Ovarian cancer (C56)	–2.94E-08 (–9.89E-08, 4.01E-08)	0.403	–2.28E-03 (–4.79E-03, 2.20E-04)	0.073	–1.17E-01 (–2.21E-01, –1.27E-02)	0.028
Prostate cancer (C61)	–1.32E-07 (–1.85E-07, –7.96E-08)	<0.001	–1.98E-03 (–4.13E-03, 1.66E-04)	0.070	–1.06E-01 (–1.95E-01, –1.70E-02)	0.020
Bladder cancer (C67)	–7.34E-08 (–1.38E-07, –8.87E-09)	0.026	–2.39E-03 (–4.76E-03, –1.18E-05)	0.049	–1.35E-01 (–2.33E-01, –3.73E-02)	0.007
Cancer of the meninges, brain, spinal cord, cranial nerves, and other parts of the central nervous system (C70–72)*	–4.11E-08 (-1.74E-07, 9.16E-08)	0.539	–8.38E-03 (–1.29E-02, –3.86E-03)	<0.001	–1.65E-01 (–3.66E-01, 3.55E-02)	0.105
Thyroid cancer (C73)*	–7.81E-08 (–1.19E-07, –3.75E-08)	0.000248	–1.02E-03 (–2.61E-03, 5.68E-04)	0.205	–3.81E-03 (–7.11E-02, 6.35E-02)	0.911
All cancers combined	–5.83E-10 (–4.98E-08, 4.86E-08)	0.981	–1.18E-03 (–2.96E-03, 6.03E-04)	0.192	–9.72E-02 (–1.70E-01, –2.47E-02)	0.00919

C – cancer based on International Classification of Diseases descriptive coding, CI – confidence interval

*For every one-unit increase in risk factor, MIR is expected to decrease by the value of the coefficient.

**Figure 2:**
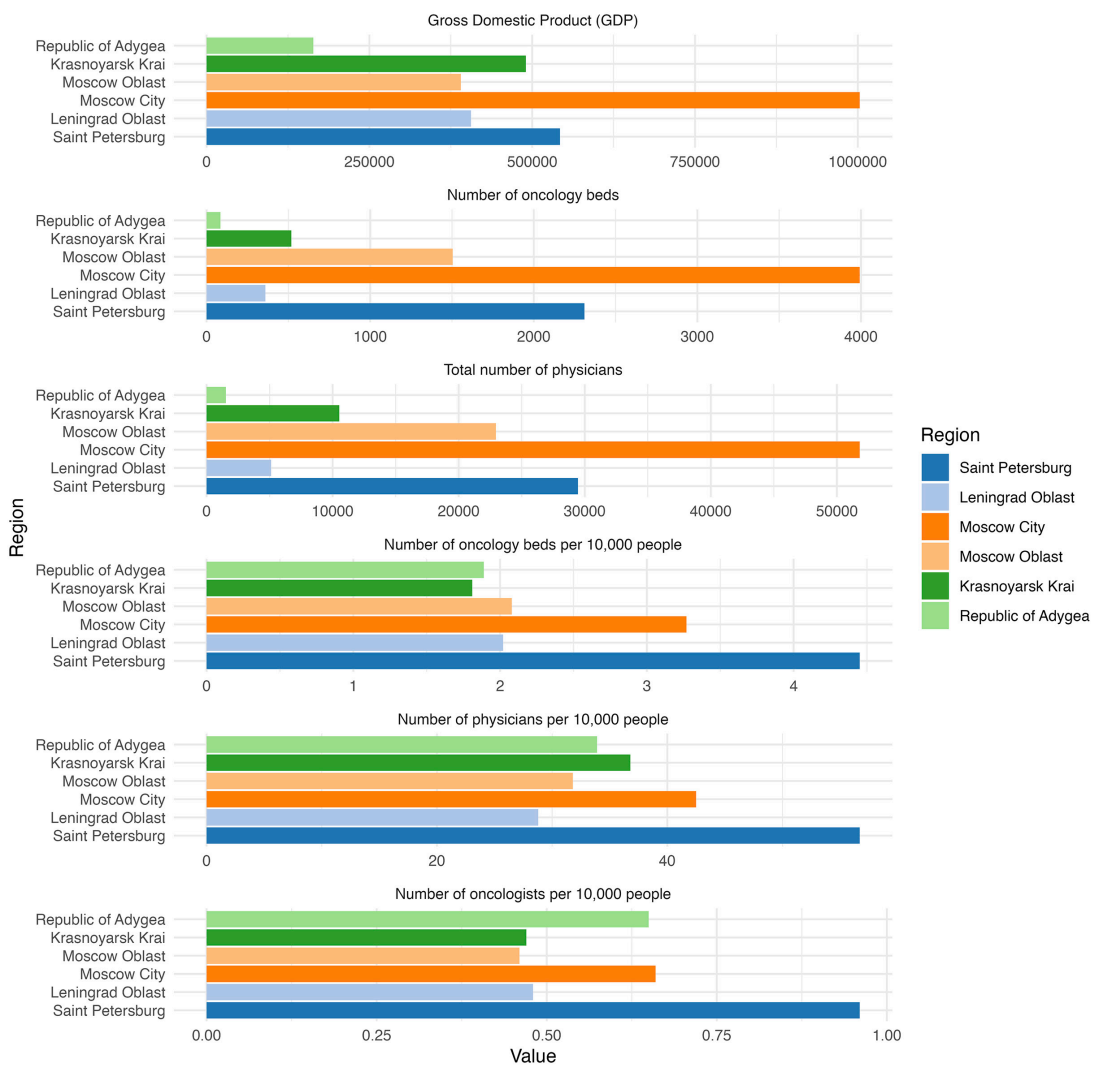
Comparison of the GDP, number of physicians, number of physicians per 10 000 people, number of oncologists per 10 000 people, and number of oncology beds across the jurisdictions of interest.

The number of physicians per 10 000 people was negatively associated with MIRs for the following cancers: colon cancer (*P* = 0.028), cancers of the rectum, rectosigmoid junction and anus (*P* = 0.032), liver and intrahepatic bile duct cancers (*P* = 0.013), melanoma (*P* = 0.029), breast cancer (*P* = 0.001), bladder cancer (*P* = 0.049), and cancers of the meninges, brain and spinal cord and other CNS parts (*P* < 0.001). Despite having one of the country’s highest numbers of physicians per 10 000 individuals (56.7 per 10 000), Saint Petersburg had some of the highest MIRs for cancers that have effective early detection/treatment approaches, such as colon, breast, cervical cancer, and melanoma (**Figure 2**).

The number of oncologists for adults per 10 000 people had a significant negative association with MIRs of stomach cancer (*P* = 0.004), colon cancer (*P* = 0.016), cancers of the rectum, rectosigmoid junction and anus (*P* < 0.001), laryngeal cancer (*P* = 0.036), cancers of the trachea, bronchi and lung (*P* = 0.024), breast cancer (*P* = 0.015), ovarian cancer (*P* = 0.028), prostate cancer (*P* = 0.02), bladder cancer (*P* = 0.007), and all cancers combined (*P* = 0.009), as expected.

## DISCUSSION

In this study, we evaluated the MIR across 20 cancer groups over 11 years in a geographically diverse region, the Russian Federation, analysing factors including GDP, physician access, and oncologist availability. MIR serves as a reliable marker for five-year survival, registry completeness, and cancer care quality. Lower MIR can be achieved with comprehensive cancer screening programs, patient education/support, early diagnosis, and access to effective treatments [16]. Specifically, a study on colorectal cancer (CRC) showed that MIR was a useful indicator for cancer screening and care and in identifying regional disparities overall, since CRC is one of the malignancies where screening and early detection are possible [15]. Positive correlation between MIR values and healthcare system rankings has also been identified [15]. Another study showed that MIR appears to be an accurate predictor of five-year survival rates, and several studies demonstrated that the MIR is a useful measure of the completeness of a cancer registry, especially when considering the quality of cancer care and reporting in a country [17]. MIR has been used to describe cancer disparities in African Americans *vs.* Caucasians in South Carolina, and further studies are focusing on examining associations with additional attributes of the healthcare system [18,19]. On the other hand, for cancers that do not have effective treatment or early detection strategies, such as pancreatic cancer, no correlation has been noted to health expenditures [20].

For Russia overall, MIR for liver, intrahepatic bile duct cancer and pancreatic cancer are >1, reflecting limitations in early diagnosis and supporting GLOBOCAN 2022 data ranking Russia’s liver and intrahepatic bile duct cancer (MIR = 0.963) as fourth highest among Organisation for Economic Co-operation and Development (OECD) countries, surpassed by Chile (MIR = 0.976), Turkey (MIR = 0.983) and Costa Rica (MIR = 0.988) [21] (Table S3 in the **Online Supplementary Document**). Prevalence of smoking and alcohol consumption in Russia, significant risk factors for these malignancies, are among the highest globally, including in OECD countries and may explain these findings [22]. Indeed, previous reports for the 2019 Russian Federation cancer statistics indicate a MIR of 1.09 for liver cancer in individuals consuming alcohol. Studies have shown that alcohol consumption, regardless of amount, is associated with decreased healthcare utilisation [23]. This suggests possible barriers to care inherent to alcohol consumption, including a lack of self-motivation or capacity to seek care, leading to delayed diagnosis and consequently worse outcomes, leading to high mortality. Thus, high smoking and alcohol consumption rates, significant risk factors, are likely contributing to these findings [23].

When compared to MIR in capital cities (Moscow and Saint Petersburg), the surrounding regions (*i.e.* oblast) consistently observed higher MIRs. This analysis was only possible because both Moscow and Saint Petersburg have distinct status as Subjects of the Russian Federation, as capital cities with a special status. However, we anticipate that similar trends would apply to other major regional capital cities *vs.* surrounding regions in Russia. It is possible that people in surrounding regions may not have access to more comprehensive care, leading to an increased MIR. It may also reflect relatively lower socioeconomic status in the surrounding regions, as evidenced by the GDP (**Figure 2**). This trend was consistent for Moscow city *vs.* Moscow Oblast and Saint Petersburg *vs.* Leningrad Oblast. In fact, Leningrad Oblast was one of the worst performers with respect to both preventable/treatable cancers and those that are not, suggesting a general lack of access to the healthcare system in the region, despite being among the wealthier jurisdictions alongside the Moscow Oblast. Moscow Oblast had the highest MIR for colon cancer at 0.76, whereas Moscow City was lower (MIR = 0.72), indicating better screening/treatment. It is important to note that Moscow and Leningrad Oblast ranked first and third nationally for the influx of migrants, ranked by the number of people moving to these jurisdictions for economic opportunities within the Russian Federation, which could impact the MIR.

Notably, the Republic of Adygea, a small jurisdiction (ranking in the bottom 10 in terms of population), is completely geographically enveloped by the Krasnodar krai. The Republic of Adygea had elevated MIR for preventable cancers such as sigmoid, rectal, and anal cancer, lip and oropharyngeal cancer, lung cancer, breast cancer, cervical cancer, and melanoma. Whereas Krasnodar krai performed significantly better and showed overall trends similar to those of other regions in the country, without notable outliers. The Republic of Adygea is an ethnically and culturally distinct region of the Russian Federation, with <500 000 residents (25% of whom are Circassian people) and is one of the poorest regions of Russia. Hence, these unique cultural, economic, and geographic factors are likely to contribute to higher MIR in the jurisdiction. Krasnodar krai has a higher GDP and a greater number of physicians and physicians per 10 000 of the population, as well as oncology beds. The only indicator investigated that was higher in the Republic of Adygea was the number of oncologists per 10 000 people, although the overall number is very low (0.65 per 10 000 people) (**Figure 2**).

It has been well demonstrated that the rate of cancer screening is lower, and the mortality is higher in rural regions. A study in Europe found significant disparities in CRC screening uptake and associated factors, including educational level, household income, and rural living status [24]. In Canada, cancer was the leading cause of death in females in rural regions, and the second leading cause in males [25]. A study on CRC in Virginia, USA, found that lower socioeconomic index and rurality were associated with higher MIR [26].

Interestingly, in major capital cities such as Moscow and Saint Petersburg, we observed an increased melanoma incidence in our prior studies [4,27], which is consistent with the higher MIR reported in this report. This is fitting as the risk factors for melanoma include intense intermittent sun exposure, and in a country with a temperate climate, this is associated with increased travel to sunny vacations and affluence [4,5,27,28]. An increase in melanoma MIR was not observed in surrounding regions despite being at the same latitude/longitude, indicating that socioeconomic factors and access to medical care and skin surveillance play a role [29]. In fact, there was a positive trend in relation to GDP, indicating that more affluent regions may have an increased burden of melanoma. Socioeconomic status (SES) has been well-demonstrated to be associated with higher melanoma incidence and lower mortality [30–32]. Sadly, despite the ability to detect and treat this cancer at early stages, MIRs in Russia seem to correlate with incidence trends. A study in the UK and Canada found a higher incidence of melanoma among higher SES groups, possibly due to exposure to UV radiation through travel or other behavioural differences [33]. In the USA, it was found that counties with lower poverty and better education, employment and income had higher age-adjusted melanoma incidence rates for early disease [32].

We found that cancers that can be detected early through screening and are treatable, such as colon and anal cancer, melanoma, breast, and bladder cancer, had a favourable association with the number of physicians per 10 000 individuals, suggesting limited healthcare access impacts MIR. However, the opposite was not always the case as Saint Petersburg ranked second by number of physicians per 10 000 individuals, yet there was a high rate of preventable cancers of the breast, cervical, colon and melanoma, which requires further investigation. This could, in part, be explained by the migration of the population to the capital city from other regions.

GDP had a significant negative association with MIR for breast, prostate, bladder, and thyroid cancers that can be detected early and treated, as expected. Similar trends have been observed in the literature, in which the overall MIR in high-income countries (MIR = 0.47) was significantly lower than that in middle/low-income countries (MIR = 0.64). While MIR for combined/all malignancies has decreased in recent years in the Russian Federation to 0.49 in 2022, it still ranks 10th among OECD countries based on GLOBOCAN reports (Table S3 in the **Online Supplementary Document**). In high-income countries, GDP and health expenditure showed significant inverse correlations with overall cancer MIR [34]. In the literature, other preventable cancers, including colon and breast, show a significant inverse association with GDP. Specifically, higher wealth and health expenditures in European countries were associated both with increased cancer incidence and decreased cancer mortality. For CRC, lower MIRs significantly correlated with higher current health expenditure as a percentage of GDP (*P* < 0.001) [6]. Interestingly, for pancreatic cancer (MIR = 1.01), a study across 57 countries did not find an association with GDP [35], which is consistent with our study. This may be explained by the fact that this malignancy is not easily detected early or treated.

Overall, it was surprising that very few regions have consistently performed poorly across several cancers, indicating these are likely non-random findings (**Table 1**; Figures S1–9 and Table S2 in the **Online Supplementary Document**). This highlights the regional disparities in cancer screening and access to treatment, particularly when comparing MIRs between capital cities and surrounding jurisdictions (**Figure 1**). For regions that consistently demonstrated lower MIRs, including the Republic of Ingushetia and the Chechen Republic, we cannot rule out the possibility that MIRs are artificially low due to political unrest or other factors affecting cancer reporting in these jurisdictions.

Previous studies in the USA and India have similarly shown rural-urban diagnostic disparities, exhibiting similar or lower cancer incidence in rural regions but higher mortality, particularly in cancers with effective interventions and detection strategies [36,37]. It is likely that when other countries are examined at a more granular level, similar trends will emerge. Hence, while this data are specific to the Russian Federation, the highlighted trends are likely applicable to other countries. Future studies are required to evaluate MIR in other countries/jurisdictions and focus on the implementation of resources in the resource-poor areas.

This is a retrospective study based on open-source national data from the Russian Federation population during 2008–2018. The data used in this study was retrieved from a large national database, and given this approach, bias may arise if inaccuracies/errors exist in data reported by individual jurisdictions. However, mandatory reporting is required in the country, and large patient numbers minimise the risk of bias from this approach. The cases are recorded in the registry in accordance with IARC requirements for the gathering and analysis of population data. This study uses an ecological design and precludes assessment of individual-level risk factors, and as with all ecological studies, population-level associations may not reflect individual outcomes. Information about the cancer subtype, stage, and treatment approaches was not available for the study. The lack of control for environmental and behavioural risk factors, including smoking, alcohol use, and occupational exposures, as well as the exclusion of data from recent years affected by the COVID-19 pandemic and geopolitical events, limits the generalizability of our findings. Data access limitations prevented the annual calculation of rates, and crude rather than age-standardised rates were applied. We also note that the number of oncologists in our study (approximately 0.5 per 10 000 population) was relatively high compared to previously published literature. One study estimates that global clinical oncologists are available at a rate of 0.028 per 100 000 people [38]. Finally, MIR was used as a proxy for cancer survival in this study, which may not fully capture the complexity of the disease and its management and can vary if there are inaccuracies in the reporting of incidence and/or mortality. It is an indirect measure that may be influenced by underdiagnosis and reporting errors, particularly where MIR>1.

## CONCLUSIONS

In this study, we identified significant disparities in MIR for cancer between capital cities and their surrounding jurisdictions. Moreover, we discovered that the Republic of Adygea, which is historically and culturally distinct from the surrounding regions, has several poor indicators compared to the Krasnodar krai region that geographically encompasses this jurisdiction. More affluent regions were affected by melanoma, whereas surrounding regions with similar climate and UV exposure did not have this signal. Of all the cancers studied, we expected to see more random associations. However, instead, we consistently observed certain regions having high MIR. We suspect that similar trends (city/capital *vs.* surrounding rural region) can be found in other countries/regions. Hence, further study in this area is needed to minimise health disparities.

## Additional material


Online Supplementary Document
