# Supplementary material for: Populational analysis of the mortality-to-incidence ratio across 20 cancer groupings in the Russian Federation
Source: J Glob Health. 2026 Mar 13;16:04084. doi: 10.7189/jogh.16.04084 (PMC12981739; doi:10.7189/jogh.16.04084)
Supplement: Online Supplementary Document [file jogh-16-04084-s001.pdf]

Supplement to: Muntyanu A, Moustaqim-Barrette A, Rijal H, Nechaev V, Pastukhova E, Zubarev A, Logan J, Litivinov IV. Populational analysis of the mortality to incidence ratio across 20 cancer groupings in the Russian Federation. J Glob Health. 2026;16:04084.

Supplementary Figure 1. Regression curve for 9 cancer groupings where Leningrad oblast is an outlier (i.e., beyond the 95% CI). The crude mortality rate is on the y-axis and crude incidence is on the x-axis. Dotted lines indicate the 95% Confidence Interval. Cancer groupings included were: A) all sites, B) esophagus, C) stomach, D) colon, E) liver, F) pancreas, G) brain/CNS, H) ovaries and I) tracheal, bronchial, and lung cancer.

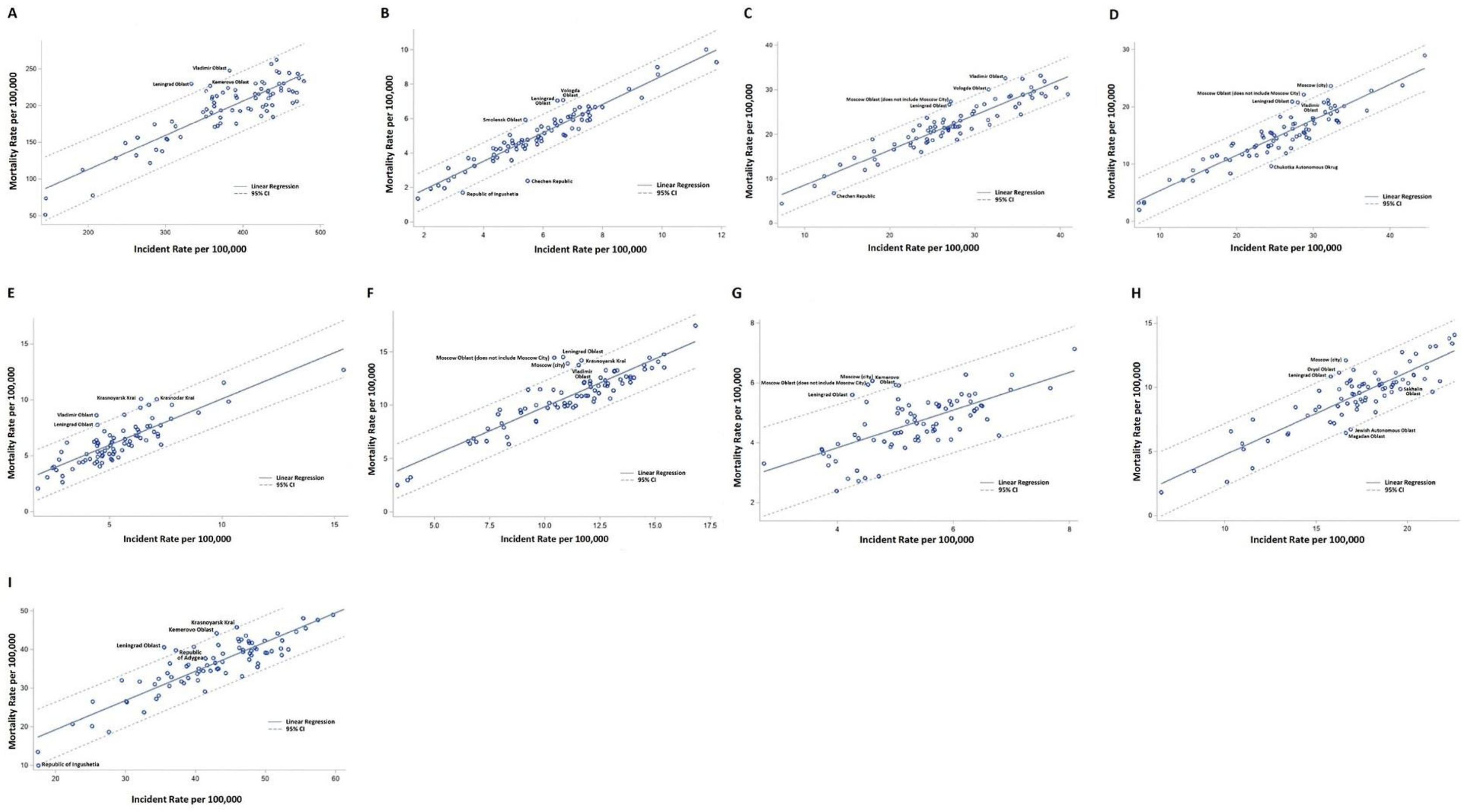

Supplementary Figure 2. Regression curve for 4 cancer groupings where Moscow oblast is an outlier (i.e., beyond the 95% CI). Crude mortality rate is on the y-axis and crude incidence is on the x-axis. Dotted lines indicate the 95% Confidence Interval. Cancer groupings included were: A) stomach, B) colon, C) pancreas and D) brain/CNS.

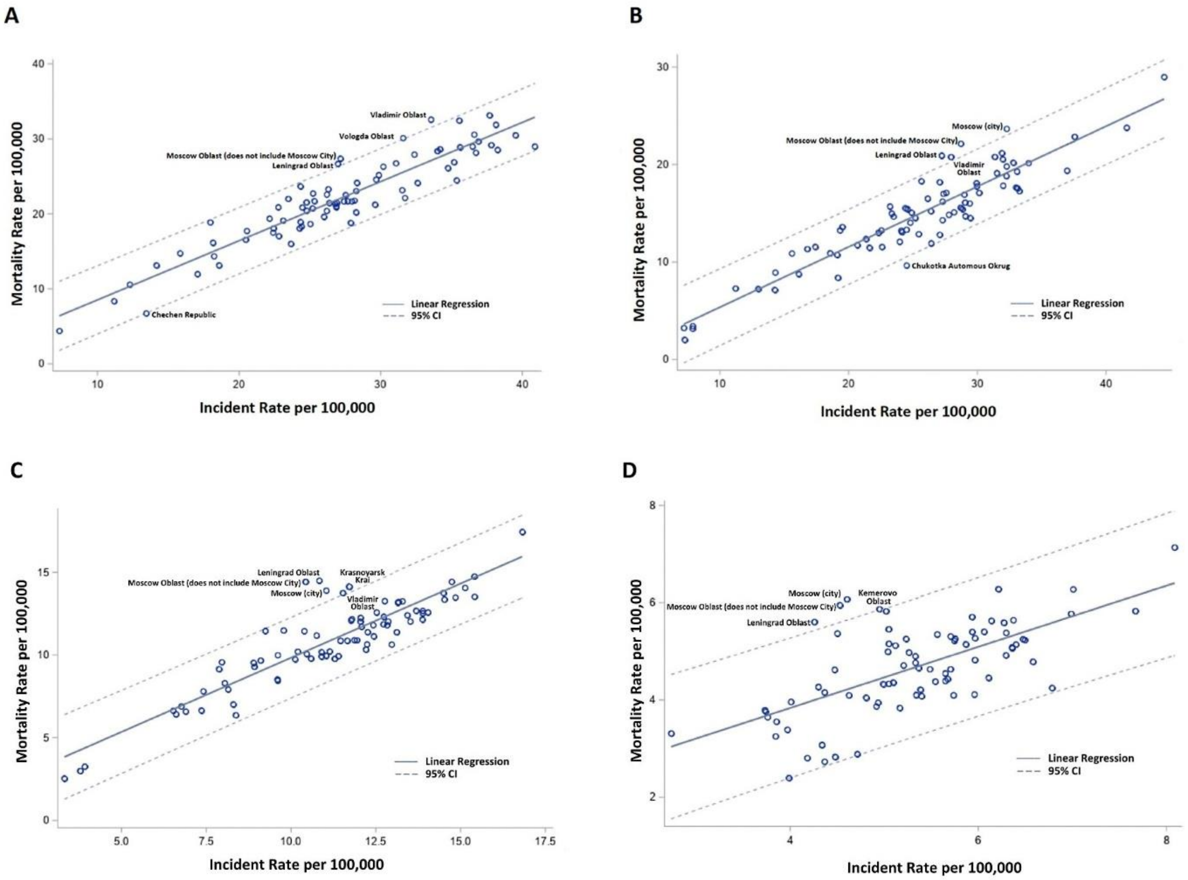

Supplementary Figure 3. Regression curve for 7 cancer groupings where Vladimir oblast is an outlier (i.e., beyond the 95% CI). Crude mortality rate is on the y-axis and crude incidence is on the x-axis. Dotted lines indicate the 95% Confidence Interval. Cancer groupings included were: A) all sites, B) stomach, C) colon, D) liver, E) pancreas, F)
breast and G) thyroid.

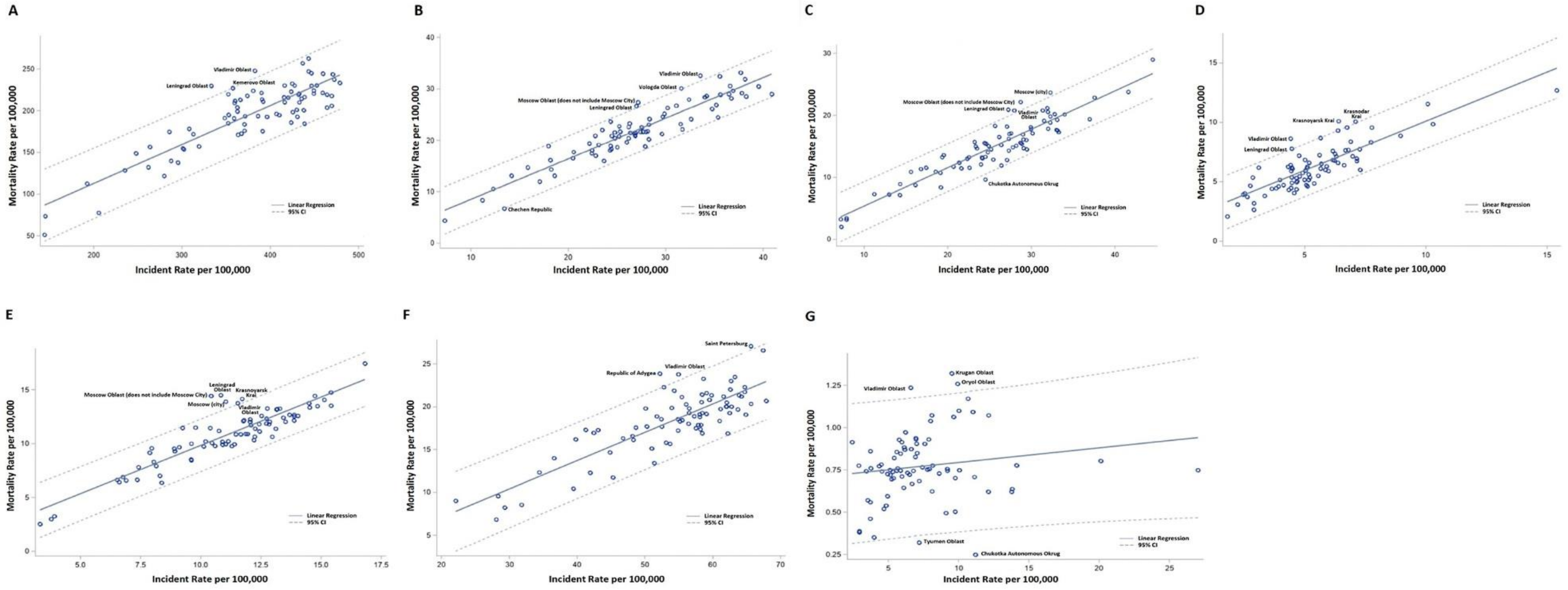

Supplementary Figure 4. Regression curve for 5 cancer groupings where Kemerovo oblast is an outlier (i.e., beyond the 95% CI). Crude mortality rate is on the y-axis and crude incidence is on the x-axis. Dotted lines indicate the 95% Confidence Interval. Cancer groupings included were: A) all sites, B) rectal, rectosigmoid, anal cancer, C) tracheal, bronchial, and lung cancer, D) bones and sarcoma and E)

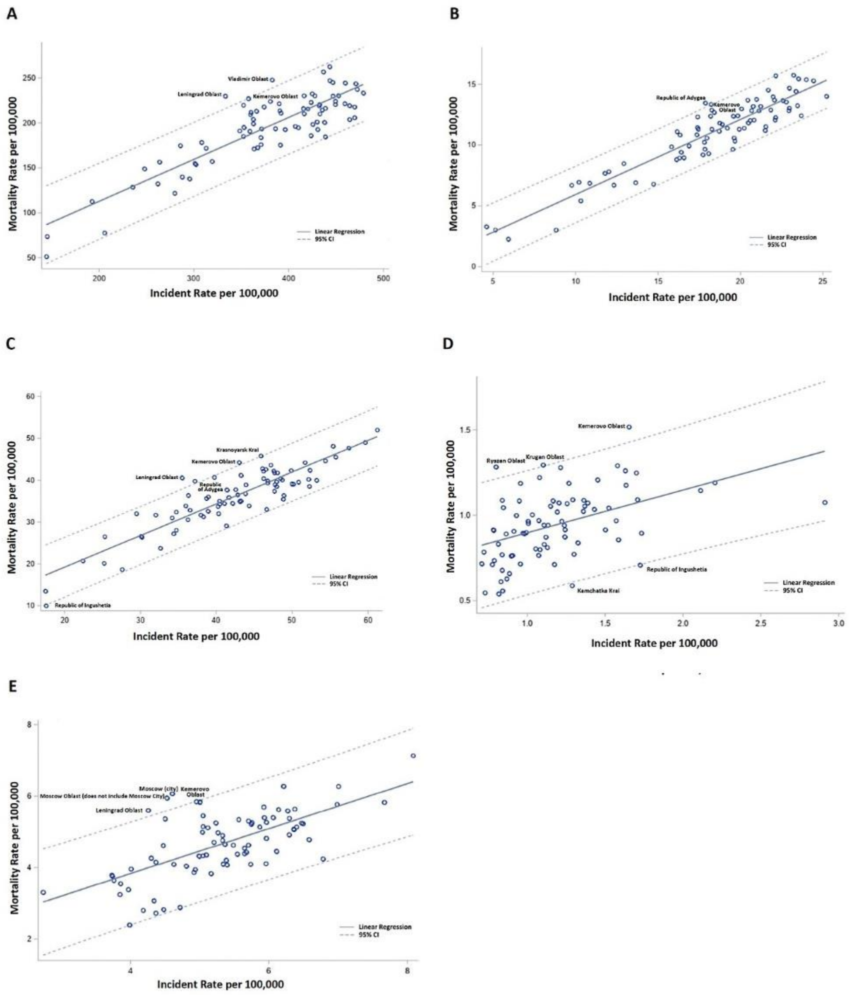

Supplementary Figure 5. Regression curve for 3 cancer groupings where Krasnoyarsk oblast is an outlier (i.e., beyond the 95% CI). Crude mortality rate is on the y-axis and crude incidence is on the x-axis. Dotted lines indicate the 95% Confidence Interval. Cancer groupings included were: A) liver, B) pancreas and C) tracheal, bronchial, and lung cancer.

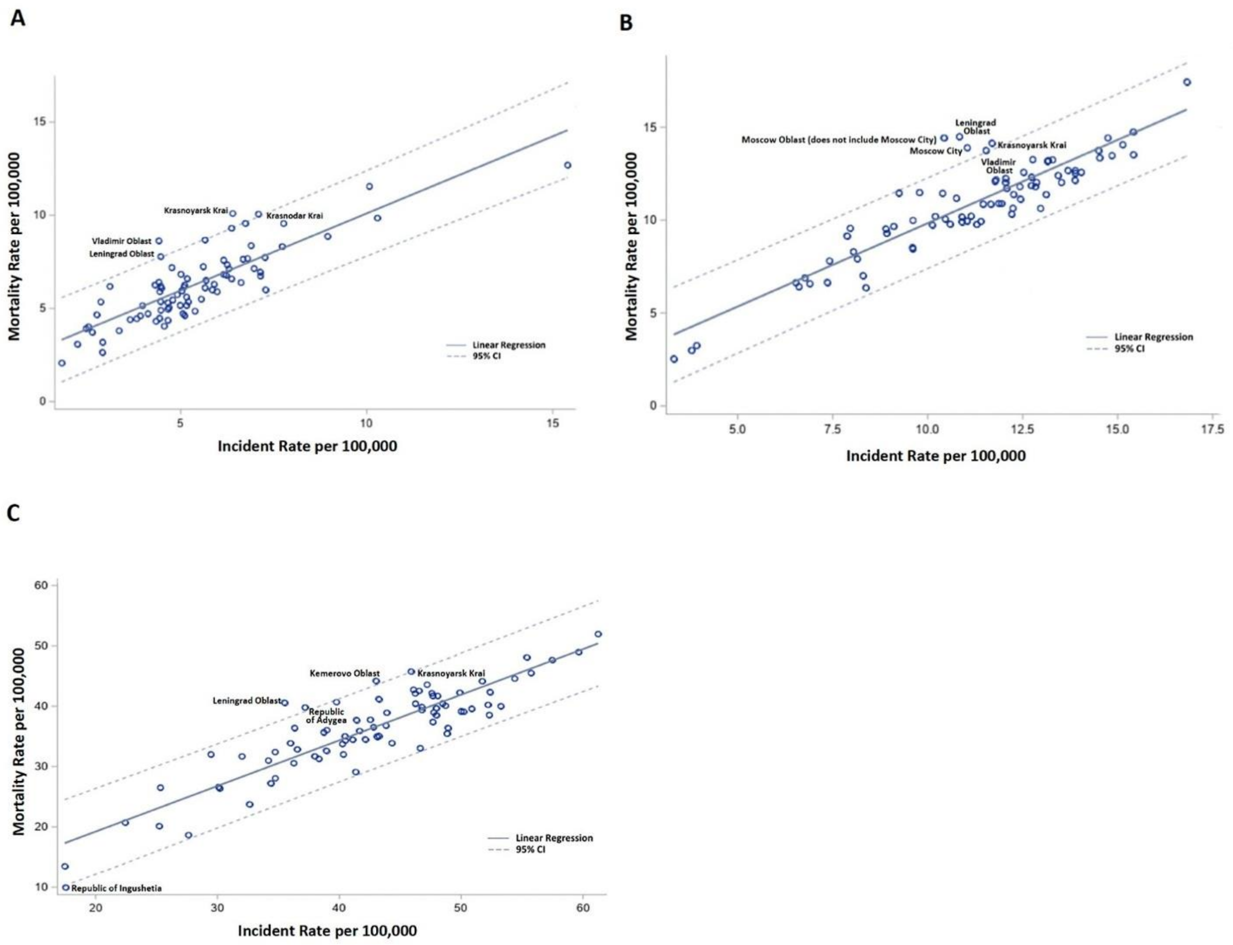

Supplementary Figure 6. Regression curve for 2 cancer groupings where Smolensk oblast is an outlier (i.e., beyond the 95% CI). Crude mortality rate is on the y-axis and crude incidence is on the x-axis. Dotted lines indicate the 95% Confidence Interval. Cancer groupings included were: A) oral and B) esophagus.

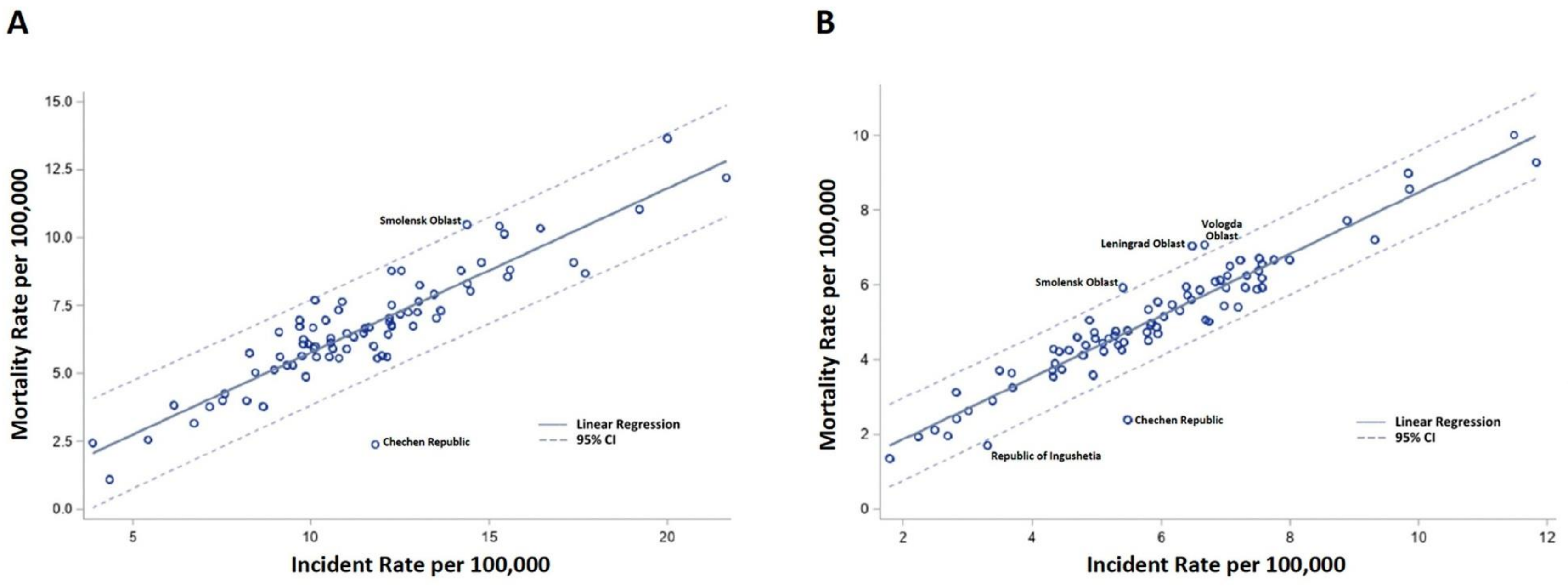

Supplementary Figure 7. Regression curve for 2 cancer groupings where Vologda oblast is an outlier (i.e., beyond the 95% CI). Crude mortality rate is on the y-axis and crude incidence is on the x-axis. Dotted lines indicate the 95% Confidence Interval. Cancer groupings included were: A) esophagus and B) stomach.

A

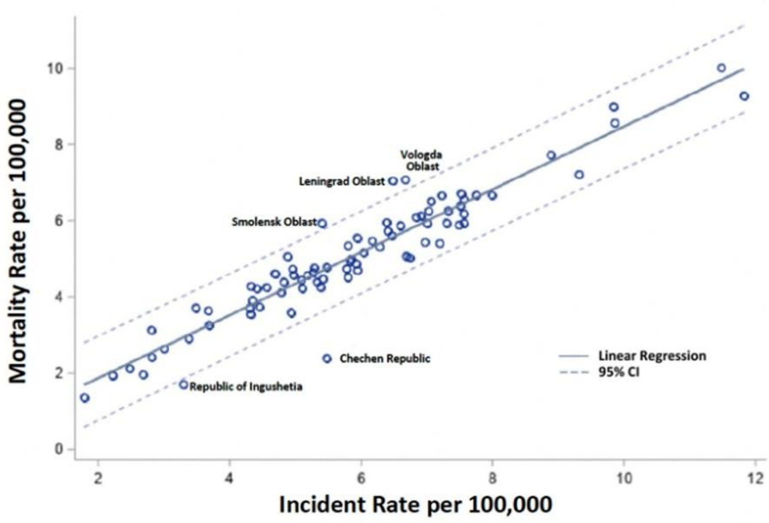

B

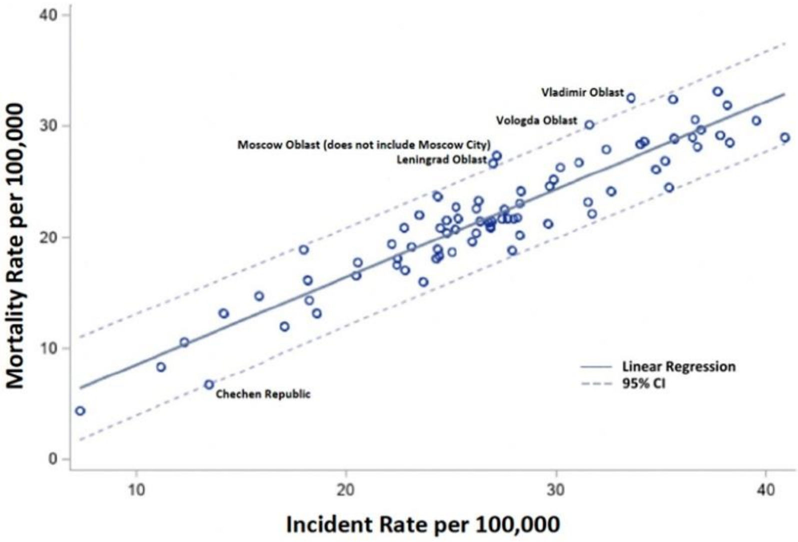

Supplementary Figure 8. Regression curve for 4 cancer groupings where Moscow City is an outlier (i.e., beyond the 95% CI). The crude mortality rate is on the y-axis and crude incidence is on the x-axis. Dotted lines indicate the 95% Confidence Interval. Cancer groupings included were: A) colon, B) pancreatic, C) cutaneous melanoma and D) ovarian cancer.

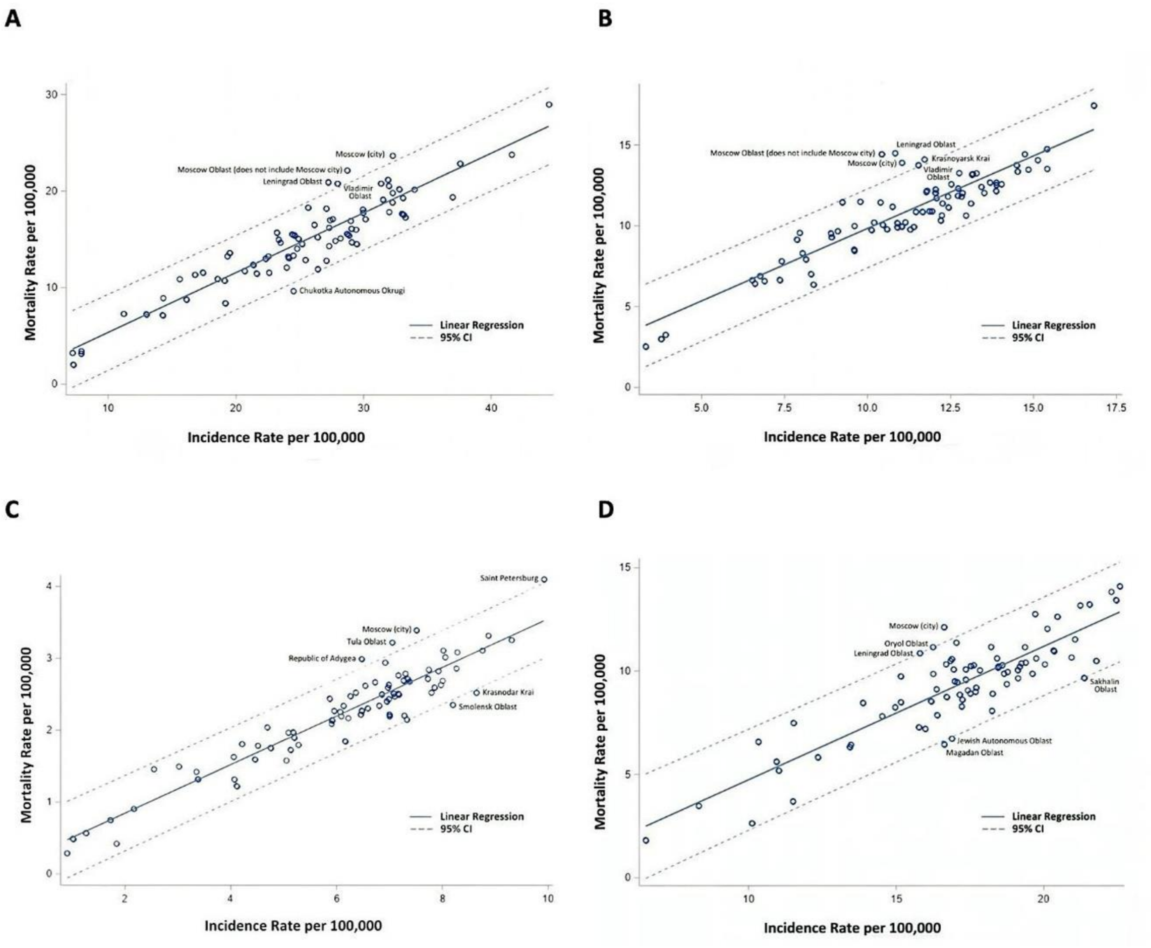

Supplementary Figure 9. Regression curve for 4 cancer groupings where the Republic of Adygea is an outlier (i.e., beyond the 95% CI). Crude mortality rate is on the y-axis and crude incidence is on the x-axis. Dotted lines indicate the 95% Confidence Interval. Cancer groupings included were: A) breast cancer, B) melanoma, C) rectal, rectosigmoid, anal cancer and D) tracheal, bronchial, and lung cancer.

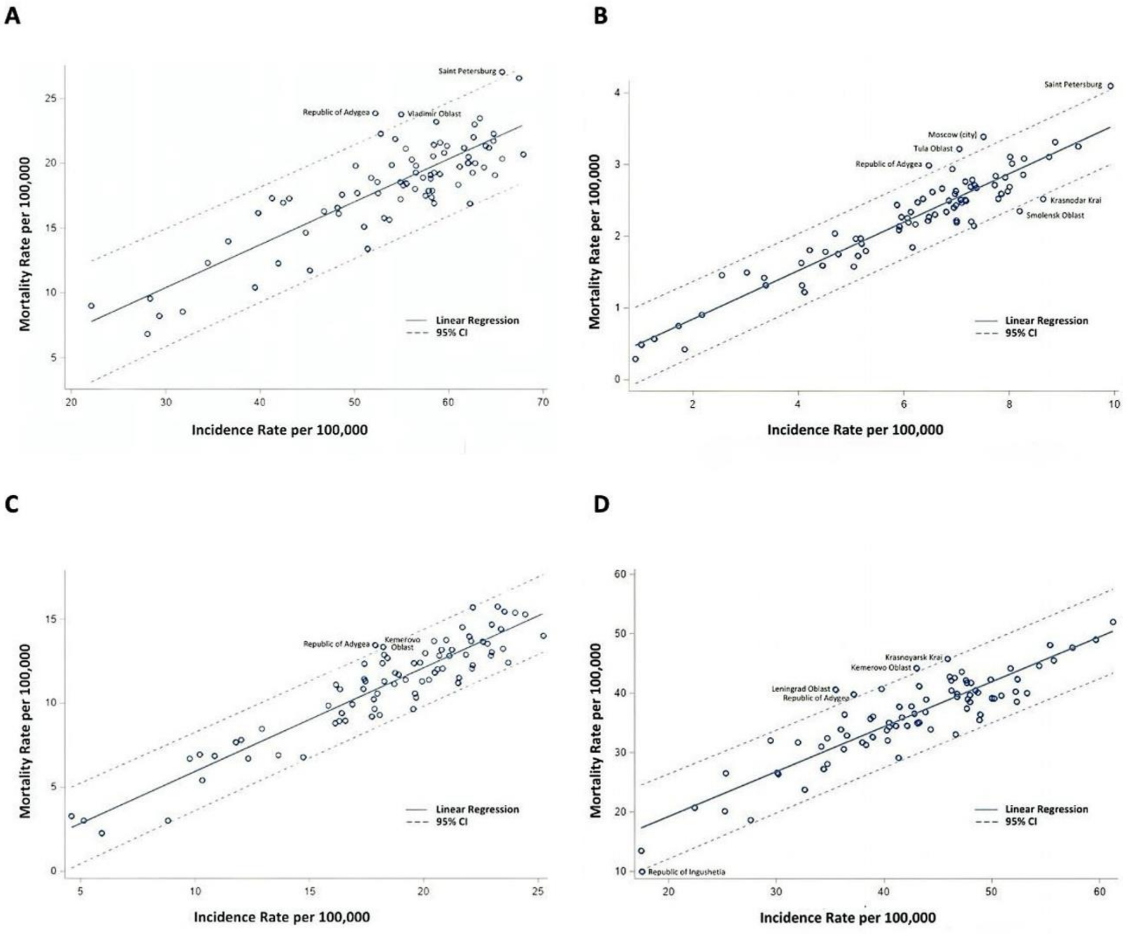
A
Saint Petersburg
Republic of Adygea
Vladimir Oblast
Mortality Rate per 100,000
Incidence Rate per 100,000
Linear Regression
95% CI
B
Saint Petersburg
Moscow (city)
Tula Oblast
Republic of Adygea
Krasnodar Krai
Smolensk Oblast
Mortality Rate per 100,000
Incidence Rate per 100,000
Linear Regression
95% CI
C
Republic of Adygea
Kemerovo Oblast
Mortality Rate per 100,000
Incidence Rate per 100,000
Linear Regression
95% CI
D
Krasnoyarsk Krai
Kemerovo Oblast
Leningrad Oblast
Republic of Adygea
Republic of Ingushetia
Mortality Rate per 100,000
Incidence Rate per 100,000
Linear Regression
95% CI

Supplementary Table 1. International Classification of Diseases-10 (ICD-10) codes and their corresponding cancer groupings.

ICD -10 Code	Cancer type
C00-14	Malignant neoplasms of the lip, oral cavity, and pharynx
C15	Esophageal Cancer
C16	Stomach Cancer
C18	Colon Cancer
C19-21	Rectosigmoid junction, rectum, and anal cancer
C22	Liver and Intrahepatic Bile Duct Cancer
C25	Pancreatic Cancer
C32	Laryngeal Cancer
C33-34	Tracheal, bronchus, and lung cancer
C40-41	Cancer of the bone articular cartilage
C43	Malignant Melanoma of the skin
C44	Skin Cancer (not including melanoma)
C50	Breast Cancer
C53	Cervical Cancer
C54	Uterine Cancer
C56	Ovarian Cancer
C61	Prostate Cancer
C67	Bladder Cancer
C70-72	Cancer of the meninges, brain, spinal cord, cranial nerves, and other parts of the central nervous system
C73	Thyroid Cancer

Supplementary Table 2. Mortality to Incidence Ratio (MIR) for each cancer type and cancers combined for each jurisdiction and federal districts of the Russian Federation between 2008-2018. Select variables of jurisdiction-specific total number and number per 10,000 people of physicians and oncologists (both pediatric and adult) and Gross domestic product (GDP) over 11 years included over study period. Abbreviations: C, cancer based on International Classification of Diseases (ICD) descriptive coding. Abbreviations: C, cancer based on International Classification of Diseases (ICD) descriptive coding.

Region	GDP (11-year average)	Total number of physicians (all types of doctors)	Number of physicians per 10,000 people	Total number of oncologists (adults and pediatric)	Number of oncologists per 10,000 people (adults)	Number of oncologists per 10,000 people (paediatric)	Malignant neoplasms of the lip, oral cavity, and pharynx (C00-14)	Esophageal Cancer (C15)	Stomach Cancer (C16)	Colon Cancer (C18)	Rectosigmoid junction, rectum, and anal cancer (C19-21)	Liver and Intrahepatic Bile Duct Cancer (C22)	Pancreatic Cancer (C25)	Laryngeal Cancer (C32)	Tracheal, bronchus, and lung cancer (C33-34)	Cancer of the bone articular cartilage (C40-41)	Malignant Melanoma of the skin (C43)	Skin Cancer (not including melanoma) (C44)	Breast Cancer (C50)	Cervical Cancer (C53)	Ovarian Cancer (C56)	Prostate Cancer (C61)	Bladder Cancer (C67)	Cancer of the meninges, brain, spinal cord, cranial nerves, and other parts of the central nervous system (C70-72)	Thyroid Cancer (C73)	All cancers combined
Chukotka Autonomous Okrug	1077053	318	62.9	2	0.53	0	0.489	0.785	0.677	0.345	0.507	0.825	0.761	0.389	0.779	0.75	0.571	0.019	0.265	0.282	0.323	0.147	0.4	0.643	0.022	0.468
Saint Petersburg	542771.9	29455	56.7	446	0.96	0.31	0.681	0.935	0.86	0.648	0.617	1.147	1.038	0.635	0.922	0.832	0.413	0.039	0.413	0.53	0.625	0.408	0.445	0.861	0.088	0.588
Republic of North Ossetia	156365.9	3870	54.9	45	0.81	0.06	0.613	0.878	0.931	0.634	0.545	1.28	1.054	0.632	0.926	0.939	0.435	0.026	0.381	0.459	0.538	0.454	0.407	1.012	0.147	0.493
Magadan Oblast	673748.7	785	53	11	0.93	0	0.553	0.836	0.744	0.579	0.515	1.026	0.868	0.742	0.817	0.75	0.337	0.032	0.299	0.238	0.389	0.299	0.393	0.595	0.099	0.502
Sakha Republic (Yakutia)	661785.4	4617	48.2	43	0.57	0.12	0	0.786	0.708	0.5	0.525	0.825	0.847	0.599	0.793	0.771	0.436	0.065	0.269	0.319	0.471	0.324	0.366	0.621	0.089	0.547
Astrakhan Oblast	276657.6	4891	47.9	72	0.88	0.05	0.574	0.921	0.827	0.613	0.574	1.097	1.041	0.642	0.925	1.172	0.34	0.023	0.365	0.419	0.529	0.51	0.616	0.828	0.267	0.549
Republic of Mordovia	195347.7	3813	47.1	39	0.55	0.15	0.491	0.746	0.692	0.461	0.542	1.029	0.87	0.572	0.727	0.542	0.293	0.012	0.261	0.25	0.443	0.334	0.331	0.579	0.111	0.421
Tyva Republic	139917.2	1417	45.2	17	0.85	0	0.634	0.932	0.814	0.449	0.717	0.958	1.018	0.618	0.8	0.517	0.486	0.052	0.244	0.329	0.473	0.417	0.542	0.631	0.377	0.586
Amur Oblast	287040.6	3648	45	48	0.72	0.11	0.58	0.845	0.778	0.567	0.547	1.456	0.965	0.741	0.887	1.055	0.362	0.023	0.332	0.466	0.472	0.425	0.469	0.84	0.157	0.526
Republic of Kalmykia	165191.4	1246	44.4	13	0.61	0	0.532	0.856	0.786	0.608	0.651	0.966	0.963	0.612	0.848	0.634	0.418	0.044	0.382	0.442	0.651	0.472	0.53	0.819	0.144	0.594
Tyumen Oblast	1437157	15956	44.34	243	0.65	0.02	0.528	0.99	0.703	0.454	0.461	1.069	0.906	0.518	0.729	0.662	0.298	0.02	0.26	0.256	0.478	0.248	0.297	0.823	0.045	0.436
Yaroslavl Oblast	292836.4	5614	44.1	75	0.7	0.09	0.614	0.855	0.746	0.575	0.556	1.185	0.927	0.696	0.855	0.791	0.349	0.021	0.31	0.38	0.621	0.368	0.389	0.878	0.133	0.488
Udmurt Republic	279950.2	6639	43.8	80	0.66	0.06	0.534	0.854	0.751	0.541	0.578	0.902	0.883	0.548	0.795	0.715	0.313	0.02	0.327	0.457	0.462	0.304	0.39	0.719	0.1	0.471
Chuvash Republic	180846.5	5355	43.3	66	0.66	0.04	0.536	0.921	0.781	0.557	0.52	1.099	1.033	0.594	0.844	0.847	0.358	0.024	0.294	0.445	0.611	0.429	0.445	0.985	0.121	0.511
Sakhalin Oblast	1393079	2096	42.9	46	1.18	0	0.683	0.868	0.735	0.512	0.542	0.992	0.913	0.619	0.83	0.61	0.35	0.022	0.309	0.354	0.453	0.265	0.373	0.822	0.046	0.491
Moscow (city)	1002840	51809	42.5	738	0.66	0.33	0.696	1.067	0.938	0.714	0.688	1.88	1.26	0.662	1.048	1.017	0.452	0.029	0.371	0.529	0.73	0.274	0.473	1.321	0.12	0.589
Northwestern Federal District	438400.5	58608	42.3	793	0.67	0.12	0.636	0.92	0.848	0.637	0.618	1.209	1.04	0.66	0.916	0.824	0.384	0.031	0.369	0.405	0.595	0.388	0.45	0.919	0.093	0.568
Republic of Karelia	295519.7	2648	41.9	27	0.51	0.08	0.558	0.914	0.772	0.608	0.56	1.169	0.936	0.729	0.912	1.076	0.388	0.043	0.33	0.237	0.599	0.389	0.495	0.947	0.118	0.547
Ryazan Oblast	241858.8	4717	41.5	54	0.55	0.11	0.523	0.867	0.795	0.582	0.565	1.595	0.897	0.675	0.828	1.61	0.374	0.025	0.334	0.415	0.538	0.39	0.426	0.783	0.141	0.503

Omsk Oblast	271216.3	8106	41	116	0.71	0.08	0.616	0.824	0.779	0.57	0.568	1.069	0.877	0.542	0.771	0.72	0.354	0.017	0.323	0.428	0.551	0.307	0.358	0.763	0.045	0.468
Tomsk Oblast	381940.3	4384	40.8	60	0.69	0.05	0.615	0.779	0.716	0.515	0.62	1.147	0.933	0.625	0.842	0.725	0.366	0.021	0.301	0.378	0.509	0.29	0.371	0.83	0.127	0.505
Voronezh Oblast	263521.2	9466	40.6	111	0.55	0.08	0.632	0.886	0.825	0.599	0.649	1.391	1.039	0.592	0.813	1.024	0.382	0.011	0.333	0.426	0.516	0.343	0.435	1.028	0.072	0.475
Smolensk Oblast	228813.2	3912	40.5	54	0.67	0	0.73	1.099	0.862	0.636	0.681	1.704	1.062	0.738	0.900	0.594	0.287	0.025	0.344	0.403	0.541	0.493	0.566	0.893	0.161	0.539
Kamchatka Krai	481976.9	1286	40.5	14	0.55	0	0.566	0.751	0.745	0.508	0.575	1.092	0.866	0.604	0.839	0.457	0.3	0.028	0.29	0.347	0.488	0.274	0.324	0.645	0.064	0.471
Orenburg Oblast	337845	8055	40.3	92	0.56	0.07	0.468	0.886	0.815	0.607	0.626	1.178	1.017	0.58	0.854	0.922	0.36	0.014	0.33	0.409	0.533	0.367	0.416	1.008	0.134	0.507
Arkhangelsk Oblast	451847.4	4736	40.18	55	0.45	0.02	0.565	0.871	0.772	0.601	0.594	0.992	0.903	0.581	0.835	0.811	0.356	0.016	0.297	0.33	0.531	0.264	0.35	0.757	0.077	0.51
Far Eastern Federal District	1074567	24850	40	306	0.59	0.12	0.624	0.826	0.807	0.561	0.589	1.125	0.963	0.705	0.853	0.835	0.379	0.027	0.323	0.397	0.524	0.37	0.435	0.787	0.109	0.531
Kursk Oblast	254622.9	4466	40	67	0.73	0	0.575	0.848	0.838	0.583	0.621	1.209	0.97	0.651	0.848	1.298	0.362	0.011	0.323	0.382	0.505	0.458	0.382	0.969	0.12	0.509
Komi Republic	546924.4	3449	39.9	42	0.6	0.05	0.589	0.869	0.782	0.625	0.63	0.988	0.926	0.715	0.813	0.935	0.348	0.049	0.292	0.374	0.554	0.381	0.388	0.841	0.051	0.532
Novosibirsk Oblast	298987.9	10968	39.9	142	0.62	0.08	0.579	0.83	0.791	0.593	0.579	1.037	0.92	0.589	0.81	0.754	0.401	0.02	0.323	0.387	0.526	0.328	0.399	0.871	0.13	0.493
Khabarovsk Krai	374016.8	5343	39.9	77	0.66	0.2	0.582	0.79	0.798	0.526	0.626	1.131	0.949	0.777	0.784	0.918	0.348	0.015	0.309	0.392	0.558	0.345	0.372	0.67	0.139	0.486
Altai Republic	163499.4	851	39.8	7	0.47	0	0.471	0.99	0.892	0.609	0.681	1.083	1.019	0.75	0.933	0.541	0.369	0.047	0.358	0.469	0.638	0.517	0.589	0.833	0.106	0.602
Permsky Krai	352151.3	10298	39.1	53	0.23	0.09	0.717	0.983	0.918	0.673	0.647	1.36	1.073	0.692	0.942	0.904	0.351	0.028	0.362	0.457	0.452	0.374	0.43	0.894	0.095	0.554
Republic of Ingushetia	90702.7	1799	38.8	24	0.78	0	0.256	0.517	0.598	0.275	0.385	1.149	0.769	0.214	0.569	0.411	0.319	0.017	0.281	0.22	0.281	0.314	0.304	0.669	0.124	0.356
Kabardino-Balkaria Republic	128344.9	3279	38.1	24	0.35	0.05	0.562	0.857	0.861	0.556	0.686	0.936	0.956	0.773	0.885	0.54	0.324	0.046	0.4	0.455	0.514	0.446	0.472	0.773	0.133	0.506
Ivanovo Oblast	148000.4	3940	38	29	0.34	0	0.554	0.851	0.767	0.539	0.59	1.211	0.919	0.569	0.84	1.167	0.386	0.014	0.305	0.339	0.614	0.332	0.404	0.817	0.151	0.482
Zabaykalsky Krai	210412.7	4114	37.8	55	0.64	0.08	0.573	0.912	0.875	0.688	0.634	1.429	1.164	0.661	0.912	1.243	0.424	0.026	0.334	0.285	0.524	0.34	0.471	1.032	0.054	0.55
Siberian Federal District	316281	73005	37.8	878	0.56	0.07	0.574	0.858	0.814	0.586	0.615	1.229	0.998	0.591	0.868	0.805	0.386	0.023	0.34	0.4	0.554	0.346	0.416	0.867	0.084	0.526
Oryol Oblast	216848.2	2871	37.5	38	0.59	0.07	0.564	0.89	0.835	0.583	0.64	1.215	0.93	0.643	0.82	0.906	0.355	0.018	0.35	0.337	0.687	0.399	0.423	0.928	0.127	0.518
Samara Oblast	330254.8	12027	37.4	215	0.8	0.05	0.569	0.861	0.779	0.541	0.554	1.006	0.913	0.506	0.82	0.746	0.352	0.011	0.336	0.405	0.537	0.258	0.348	0.797	0.081	0.439
Russian Federation	389608.1	543604	37.2	7017	0.57	0.09	0.589	0.88	0.831	0.609	0.609	1.244	1.014	0.629	0.871	0.823	0.373	0.023	0.349	0.404	0.569	0.343	0.435	0.909	0.102	0.525
Saratov Oblast	213378.8	9272	37.2	86	0.41	0.04	0.463	0.793	0.755	0.511	0.563	0.909	0.848	0.508	0.709	0.763	0.348	0.011	0.319	0.379	0.52	0.323	0.344	0.757	0.134	0.439
Central Federal District	511615.1	144490	37.1	1948	0.57	0.13	0.646	0.947	0.884	0.662	0.655	1.552	1.132	0.677	0.927	0.981	0.389	0.025	0.355	0.43	0.629	0.338	0.467	1.088	0.108	0.554
Altai Krai	172744.2	8821	37	122	0.63	0.04	0.473	0.842	0.773	0.552	0.635	1.149	0.95	0.513	0.822	0.748	0.343	0.014	0.33	0.367	0.583	0.316	0.414	0.801	0.04	0.473
Republic of Buryatia	183167.5	3602	36.8	34	0.45	0.04	0.596	0.868	0.859	0.652	0.655	0.976	0.935	0.597	0.839	0.72	0.495	0.051	0.407	0.451	0.619	0.447	0.546	0.765	0.163	0.612
Krasnoyarsk Krai	490225.9	10526	36.8	114	0.47	0.12	0.577	0.954	0.886	0.628	0.658	1.578	1.193	0.688	0.998	0.856	0.381	0.033	0.33	0.414	0.553	0.366	0.413	0.853	0.129	0.588
Murmansk Oblast	435896.6	2786	36.4	41	0.65	0.07	0.577	0.779	0.675	0.548	0.539	0.889	0.871	0.513	0.705	0.736	0.324	0.015	0.294	0.392	0.585	0.226	0.375	0.755	0.052	0.449
Tver Oblast	231926.2	4769	36.3	52	0.48	0	0.619	0.89	0.837	0.621	0.641	1.148	0.957	0.753	0.869	0.92	0.33	0.03	0.352	0.435	0.53	0.371	0.435	0.886	0.119	0.55
Irkutsk Oblast	353804.8	8733	36.2	106	0.54	0.09	0.53	0.758	0.697	0.501	0.494	1.067	0.89	0.49	0.779	0.78	0.416	0.016	0.324	0.402	0.504	0.306	0.33	0.733	0.085	0.467
Volga Federal District	290011.9	107353	36.1	1357	0.55	0.05	0.546	0.838	0.785	0.568	0.58	1.057	0.928	0.598	0.812	0.822	0.359	0.019	0.332	0.384	0.53	0.327	0.398	0.829	0.119	0.488

Belgorod Oblast	381848.7	5590	36.1	89	0.69	0.04	0.577	0.873	0.862	0.585	0.596	1.599	1.203	0.673	0.84	1.238	0.303	0.019	0.325	0.425	0.5	0.386	0.344	0.925	0.11	0.482
Karachay-Cherkessia Republic	130048.8	1687	36	21	0.55	0.09	0.438	0.767	0.93	0.611	0.653	1.178	0.973	0.59	0.876	0.64	0.429	0.027	0.42	0.503	0.561	0.397	0.428	1.046	0.178	0.487
Volgograd Oblast	247356.6	9121	35.7	128	0.57	0.19	0.515	0.863	0.825	0.613	0.607	1.231	0.95	0.561	0.854	0.947	0.344	0.027	0.362	0.422	0.553	0.413	0.482	0.945	0.161	0.527
Kemerovo Oblast	290238.2	9691	35.6	87	0.39	0.05	0.669	0.957	0.974	0.673	0.733	1.539	1.175	0.654	1.029	0.918	0.386	0.041	0.423	0.463	0.644	0.459	0.523	1.246	0.103	0.636
Kirov Oblast	182538.6	4553	34.9	47	0.43	0.04	0.569	0.881	0.829	0.601	0.658	0.946	0.897	0.586	0.823	0.724	0.372	0.016	0.314	0.437	0.613	0.381	0.484	0.831	0.095	0.513
Ural Federal District	640376.6	42588	34.7	603	0.59	0.11	0.551	0.884	0.812	0.603	0.588	1.19	0.983	0.62	0.872	0.696	0.362	0.022	0.338	0.367	0.575	0.318	0.445	0.845	0.098	0.527
Republic of Bashkortostan	282404.7	13993	34.4	175	0.54	0.03	0.496	0.786	0.747	0.595	0.588	0.939	0.889	0.6	0.808	0.92	0.391	0.021	0.349	0.415	0.552	0.331	0.385	0.862	0.151	0.515
Southern Federal District	252849.7	47925	34.2	683	0.58	0.09	0.56	0.861	0.831	0.595	0.581	1.329	0.958	0.601	0.872	0.799	0.343	0.024	0.37	0.425	0.538	0.345	0.437	0.882	0.094	0.5
Republic of Adygea	164088.8	1524	33.9	23	0.65	0	0.763	1.116	1.05	0.685	0.754	1.513	1.102	0.7	1.071	1.132	0.462	0.022	0.458	0.552	0.607	0.455	0.563	1.143	0.11	0.547
North Caucasian Federal District	146766.8	32627	33.8	343	0.48	0.03	0.49	0.72	0.768	0.512	0.558	1.065	0.926	0.564	0.797	0.645	0.383	0.026	0.371	0.395	0.448	0.395	0.415	0.827	0.116	0.471
Lipetsk Oblast	321656.3	3912	33.8	68	0.69	0.1	0.552	0.826	0.718	0.511	0.526	1.103	0.904	0.482	0.745	0.664	0.317	0.014	0.305	0.368	0.563	0.33	0.329	0.771	0.111	0.448
Novgorod Oblast	303706.1	2088	33.7	34	0.67	0	0.52	0.774	0.71	0.506	0.524	1.228	0.908	0.599	0.752	0.972	0.336	0.021	0.272	0.285	0.482	0.324	0.305	0.76	0.074	0.464
Ulyanovsk Oblast	209708.1	4236	33.6	64	0.61	0	0.588	0.846	0.844	0.615	0.622	1.032	0.968	0.646	0.816	1.089	0.382	0.018	0.357	0.383	0.623	0.399	0.46	1.02	0.174	0.52
Republic of Khakassia	277268.2	1792	33.4	18	0.43	0	0.562	0.729	0.785	0.567	0.593	1.366	1.029	0.456	0.876	0.851	0.402	0.017	0.296	0.429	0.5	0.397	0.479	0.899	0.13	0.563
Krasnodar Krai	300523.6	18088	33.2	235	0.52	0.08	0.55	0.862	0.806	0.551	0.522	1.421	0.929	0.597	0.863	0.845	0.291	0.021	0.342	0.417	0.509	0.286	0.391	0.875	0.055	0.459
Bryansk Oblast	186233	4081	33.1	58	0.55	0.09	0.683	0.861	0.804	0.504	0.636	1.039	0.932	0.721	0.804	0.911	0.348	0.023	0.33	0.38	0.515	0.41	0.511	0.899	0.028	0.499
Nizhny Novgorod Oblast	294714.9	10714	32.8	122	0.42	0.14	0.537	0.815	0.765	0.541	0.568	1.209	0.92	0.657	0.765	0.761	0.35	0.027	0.309	0.369	0.539	0.364	0.413	0.801	0.147	0.493
Primorsky Krai	325636.1	6290	32.5	55	0.31	0.17	0.664	0.874	0.902	0.63	0.623	1.422	1.039	0.747	0.923	0.852	0.424	0.034	0.351	0.467	0.581	0.446	0.522	0.926	0.135	0.583
Republic of Dagestan	150590.5	9650	32.3	66	0.31	0.01	0.472	0.734	0.749	0.458	0.586	0.917	0.792	0.666	0.773	0.685	0.454	0.041	0.408	0.423	0.419	0.387	0.444	0.678	0.132	0.508
Moscow Oblast (does not include Moscow city)	390374.2	22964	31.8	286	0.46	0.08	0.72	1.037	1.009	0.759	0.708	1.999	1.385	0.749	1.088	0.993	0.377	0.033	0.366	0.398	0.668	0.37	0.546	1.252	0.115	0.623
Sverdlovsk Oblast	366510.2	13653	31.5	164	0.44	0.15	0.559	0.933	0.819	0.665	0.606	1.236	1.032	0.6	0.954	0.616	0.372	0.025	0.368	0.374	0.627	0.316	0.474	0.81	0.097	0.568
Stavropol Krai	181812.2	8766	31.3	104	0.45	0.05	0.589	0.858	0.809	0.545	0.589	1.166	1.042	0.569	0.818	0.932	0.372	0.02	0.354	0.408	0.481	0.397	0.434	0.884	0.096	0.474
Mari El Republic	186751.1	2145	31.2	15	0.27	0	0.552	0.921	0.87	0.665	0.664	0.997	0.974	0.809	0.908	0.817	0.365	0.053	0.402	0.367	0.555	0.428	0.486	0.756	0.13	0.579
Republic of Tatarstan	415643.3	12043	31.2	230	0.75	0	0.596	0.817	0.775	0.554	0.535	1.081	0.911	0.624	0.835	0.893	0.396	0.027	0.337	0.285	0.52	0.318	0.443	0.77	0.119	0.496
Chelyabinsk Oblast	282535	10872	31.1	171	0.59	0.1	0.57	0.901	0.854	0.623	0.621	1.294	1.003	0.663	0.887	0.74	0.381	0.019	0.368	0.439	0.576	0.348	0.491	0.878	0.153	0.538
Penza Oblast	199258.6	4210	31.1	73	0.64	0.04	0.525	0.821	0.741	0.553	0.546	1.014	0.924	0.586	0.786	0.851	0.367	0.012	0.338	0.378	0.509	0.252	0.328	0.923	0.153	0.438
Rostov Oblast	232999.6	13055	30.8	212	0.59	0.07	0.622	0.877	0.862	0.622	0.619	1.333	0.918	0.647	0.866	0.706	0.397	0.025	0.403	0.436	0.56	0.336	0.436	0.802	0.132	0.521
Tambov Oblast	223626.3	3230	30.4	38	0.43	0	0.566	0.823	0.753	0.492	0.584	1.142	0.82	0.643	0.738	0.773	0.331	0.026	0.307	0.374	0.595	0.376	0.419	0.784	0.14	0.485
Kaluga Oblast	301131.1	3060	30.3	55	0.66	0	0.63	0.891	0.812	0.568	0.657	1.144	1.005	0.666	0.888	0.608	0.346	0.025	0.345	0.431	0.548	0.303	0.436	0.955	0.082	0.515
Vologda Oblast	330416.7	3600	30.2	31	0.31	0.04	0.591	1.06	0.953	0.644	0.67	1.453	1.241	0.611	1.003	0.832	0.346	0.025	0.345	0.226	0.482	0.451	0.518	1.128	0.133	0.583

Kostroma Oblast	206852.7	1956	29.9	27	0.51	0	0.589	0.893	0.835	0.648	0.67	1.297	0.997	0.61	0.875	1.032	0.369	0.023	0.352	0.41	0.537	0.442	0.497	0.894	0.231	0.553
Kaliningrad Oblast	314310.6	2888	29.8	25	0.32	0	0.639	0.9	0.853	0.583	0.633	1.39	1.001	0.715	0.99	0.799	0.374	0.027	0.367	0.424	0.577	0.41	0.44	1.072	0.203	0.55
Leningrad Oblast	406067.5	5109	28.8	72	0.48	0	0.702	1.088	0.99	0.736	0.69	1.746	1.338	0.763	1.145	0.738	0.397	0.042	0.395	0.526	0.688	0.488	0.563	1.404	0.11	0.691
Pskov Oblast	183536.2	1849	28.4	20	0.37	0	0.701	0.887	0.913	0.712	0.626	1.133	1.003	0.733	0.915	0.734	0.355	0.018	0.397	0.305	0.618	0.44	0.624	1.006	0.144	0.557
Tula Oblast	258905.4	4278	28.3	63	0.49	0.04	0.658	0.894	0.88	0.683	0.678	1.109	0.979	0.744	0.928	1.145	0.456	0.04	0.394	0.452	0.621	0.442	0.558	0.94	0.11	0.593
Jewish Autonomous Oblast	239685.8	467	27.7	10	0.76	0	0.696	0.825	0.798	0.551	0.548	1.463	1	0.673	0.869	0.774	0.313	0.049	0.353	0.449	0.399	0.385	0.444	1	0.217	0.582
Vladimir Oblast	225653.4	3855	27.4	46	0.4	0	0.717	0.922	0.971	0.734	0.706	1.958	1.208	0.757	1.024	0.962	0.371	0.039	0.433	0.393	0.648	0.455	0.628	1.177	0.188	0.648
Chechen Republic	97322.6	3576	26.1	59	0.7	0	0.201	0.437	0.501	0.355	0.341	1.138	0.831	0.201	0.675	0.369	0.23	0.03	0.338	0.244	0.261	0.276	0.273	0.987	0.111	0.377
Kurgan Oblast	182385.7	2107	24.2	25	0.35	0.06	0.512	0.88	0.871	0.603	0.71	1.078	0.878	0.763	0.849	1.176	0.396	0.024	0.32	0.434	0.599	0.448	0.513	0.95	0.139	0.532

Supplementary Table 3. Mortality to Incidence Ratio (MIR) for liver and intrahepatic bile duct, pancreatic and all combined cancer types of Organization for Economic Cooperation and Development (OECD) countries in 2022. Data adapted from GLOBOCAN cancer observatory and reported in crude rate (per 100,000). Abbreviations: C, cancer based on International Classification of Diseases (ICD) descriptive coding.

OECD Country	Mortality (liver and intrahepatic bile ducts)	Incidence (liver and intrahepatic bile ducts)	Actual MIR (liver and intrahepatic bile ducts)	Mortality (pancreatic cancer)	Incidence (pancreatic cancer)	Actual MIR (pancreatic cancer)	Mortality (all cancers)	Incidence (all cancers)	Actual MIR (all cancers)
**Australia**	9.9	12.8	0.773	13.6	15.3	0.890	199.0	814.5	0.244
**Austria**	10.3	11.8	0.873	22.2	23.8	0.933	246.9	559.0	0.442
**Belgium**	9.8	11.4	0.860	18.9	20.0	0.945	248.6	695.3	0.358
**Canada**	10.8	13.0	0.831	17.0	18.1	0.939	261.7	760.9	0.344
**Chile**	8.2	8.4	0.976	9.3	9.9	0.939	163.3	311.0	0.525
**Columbia**	4.7	5.0	0.940	5.0	5.5	0.909	110.1	228.3	0.482
**Costa Rica**	8.1	8.2	0.988	6.5	6.7	0.970	117.2	257.1	0.456
**Czech Republic**	8.7	9.8	0.888	21.5	23.1	0.931	261.5	611.7	0.427
**Denmark**	10.2	11.5	0.887	20.9	23.2	0.901	294.8	837.0	0.352
**Estonia**	9.9	10.4	0.952	21.0	22.9	0.917	309.1	609.0	0.508
**Finland**	11.7	12.2	0.959	25.2	27.0	0.933	242.0	678.0	0.357
**France**	16.0	18.6	0.860	22.4	24.2	0.926	290.6	737.3	0.394
**Germany**	10.4	11.9	0.874	25.4	26.1	0.973	301.8	722.2	0.418
**Greece**	14.2	16.2	0.877	21.1	22.3	0.946	313.9	636.9	0.493
**Hungary**	9.3	10.0	0.930	23.8	25.2	0.944	338.1	690.6	0.490
**Iceland**	7.8	9.3	0.839	13.0	16.2	0.802	193.7	514.5	0.376
**Ireland**	9.9	11.2	0.884	12.3	13.8	0.891	207.9	622.3	0.334
**Israel**	3.9	4.2	0.929	12.7	13.3	0.955	144.1	341.1	0.422
**Italy**	15.9	19.7	0.807	24.7	26.1	0.946	321.4	723.9	0.444
**Japan**	21.0	33.0	0.636	34.5	37.9	0.910	339.4	800.4	0.424
**Republic of Korea**	24.5	28.8	0.851	14.8	17.3	0.855	164.6	236.8	0.695

**Latvia**	9.3	10.2	0.912	22.7	24.6	0.923	317.6	619.7	0.513
**Lithuania**	10.0	11.0	0.909	21.2	22.7	0.934	313.7	616.6	0.509
**Luxembourg**	7.8	9.8	0.796	13.7	14.8	0.926	170.2	535.5	0.319
**Mexico**	5.8	6.5	0.892	4.0	4.4	0.909	73.1	157.5	0.464
**Netherlands**	8.5	9.3	0.914	19.6	20.3	0.966	289.3	768.8	0.376
**New Zealand**	6.9	7.8	0.885	12.8	13.5	0.948	230.7	779.0	0.296
**Norway**	7.8	9.0	0.867	19.0	21.2	0.896	243.0	731.3	0.332
**Poland**	6.4	7.0	0.914	15.4	15.6	0.988	318.0	553.5	0.575
**Portugal**	15.9	17.2	0.924	20.6	21.3	0.967	332.9	686.0	0.485
**The Russian Federation**	7.8	8.1	0.963	14.2	15.0	0.947	213.8	435.9	0.490
**Slovak Republic**	9.1	9.7	0.938	17.6	18.5	0.951	277.8	566.2	0.491
**Slovenia**	16.1	18.0	0.894	20.3	22.0	0.923	332.0	693.1	0.479
**Spain**	12.0	13.8	0.870	17.5	18.9	0.926	247.4	596.6	0.415
**Sweden**	9.0	9.9	0.909	21.9	23.6	0.928	250.2	677.8	0.369
**Switzerland**	10.3	12.7	0.811	19.9	22.3	0.892	221.3	664.8	0.333
**Türkiye**	5.8	5.9	0.983	9.8	10.1	0.970	151.6	280.5	0.540
**United Kingdom**	10.7	12.0	0.892	15.7	16.6	0.946	265.4	664.2	0.400
**United States of America**	9.2	13.0	0.708	14.8	18.0	0.822	180.9	710.9	0.254

STROBE Statement—checklist of items that should be included in reports of observational studies

	Item No	Recommendation	Page
**Title and abstract**	1	(*a*) Indicate the study's design with a commonly used term in the title or the abstract	1
		(*b*) Provide in the abstract an informative and balanced summary of what was done and what was found	2
**Introduction**			
Background/rationale	2	Explain the scientific background and rationale for the investigation being reported	2-3
Objectives	3	State specific objectives, including any prespecified hypotheses	3
**Methods**			
Study design	4	Present key elements of study design early in the paper	4
Setting	5	Describe the setting, locations, and relevant dates, including periods of recruitment, exposure, follow-up, and data collection	3
Participants	6	(*a*) *Cohort study*—Give the eligibility criteria, and the sources and methods of selection of participants. Describe methods of follow-up<br>*Case-control study*—Give the eligibility criteria, and the sources and methods of case ascertainment and control selection. Give the rationale for the choice of cases and controls<br>*Cross-sectional study*—Give the eligibility criteria, and the sources and methods of selection of participants	4
		(*b*) *Cohort study*—For matched studies, give matching criteria and number of exposed and unexposed<br>*Case-control study*—For matched studies, give matching criteria and the number of controls per case	-
Variables	7	Clearly define all outcomes, exposures, predictors, potential confounders, and effect modifiers. Give diagnostic criteria, if applicable	4
Data sources/ measurement	8*	For each variable of interest, give sources of data and details of methods of assessment (measurement). Describe comparability of assessment methods if there is more than one group	4-5
Bias	9	Describe any efforts to address potential sources of bias	4
Study size	10	Explain how the study size was arrived at	3
Quantitative variables	11	Explain how quantitative variables were handled in the analyses. If applicable, describe which groupings were chosen and why	4
Statistical methods	12	(*a*) Describe all statistical methods, including those used to control for confounding	4
		(*b*) Describe any methods used to examine subgroups and interactions	-
		(*c*) Explain how missing data were addressed	-
		(*d*) *Cohort study*—If applicable, explain how loss to follow-up was addressed<br>*Case-control study*—If applicable, explain how matching of cases and controls was addressed<br>*Cross-sectional study*—If applicable, describe analytical methods taking account of sampling strategy	-
		(*e*) Describe any sensitivity analyses	4

Continued on next page

**Results**			
Participants	13*	(a) Report numbers of individuals at each stage of study—eg numbers potentially eligible, examined for eligibility, confirmed eligible, included in the study, completing follow-up, and analysed	5
		(b) Give reasons for non-participation at each stage	-
		(c) Consider use of a flow diagram	-
Descriptive data	14*	(a) Give characteristics of study participants (eg demographic, clinical, social) and information on exposures and potential confounders	5
		(b) Indicate number of participants with missing data for each variable of interest	-
		(c) *Cohort study*—Summarise follow-up time (eg, average and total amount)	-
Outcome data	15*	*Cohort study*—Report numbers of outcome events or summary measures over time	-
		Case-control study—Report numbers in each exposure category, or summary measures of exposure	-
		Cross-sectional study—Report numbers of outcome events or summary measures	5
Main results	16	(*a*) Give unadjusted estimates and, if applicable, confounder-adjusted estimates and their precision (eg, 95% confidence interval). Make clear which confounders were adjusted for and why they were included	5-6
		(*b*) Report category boundaries when continuous variables were categorized	-
		(*c*) If relevant, consider translating estimates of relative risk into absolute risk for a meaningful time period	-
Other analyses	17	Report other analyses done—eg analyses of subgroups and interactions, and sensitivity analyses	-
**Discussion**			
Key results	18	Summarise key results with reference to study objectives	10
Limitations	19	Discuss limitations of the study, taking into account sources of potential bias or imprecision. Discuss both direction and magnitude of any potential bias	7-8, 11
Interpretation	20	Give a cautious overall interpretation of results considering objectives, limitations, multiplicity of analyses, results from similar studies, and other relevant evidence	7-11
Generalisability	21	Discuss the generalisability (external validity) of the study results	11
**Other information**			
Funding	22	Give the source of funding and the role of the funders for the present study and, if applicable, for the original study on which the present article is based	12

*Give information separately for cases and controls in case-control studies and, if applicable, for exposed and unexposed groups in cohort and cross-sectional studies.

Note: An Explanation and Elaboration article discusses each checklist item and gives methodological background and published examples of transparent reporting. The STROBE checklist is best used in conjunction with this article (freely available on the Web sites of PLoS Medicine at http://www.plosmedicine.org/, Annals of Internal Medicine at http://www.annals.org/, and Epidemiology at http://www.epidem.com/). Information on the STROBE Initiative is available at www.strobe-statement.org.
